# Supplementary material for: Dimension-agnostic and granularity-based spatially variable gene identification using BSP
Source: Nat Commun. 2023 Nov 14;14:7367. doi: 10.1038/s41467-023-43256-5 (PMC10645821; doi:10.1038/s41467-023-43256-5)
Supplement: Supplementary file 1 — Supplementary Information [file 41467_2023_43256_MOESM1_ESM.docx]

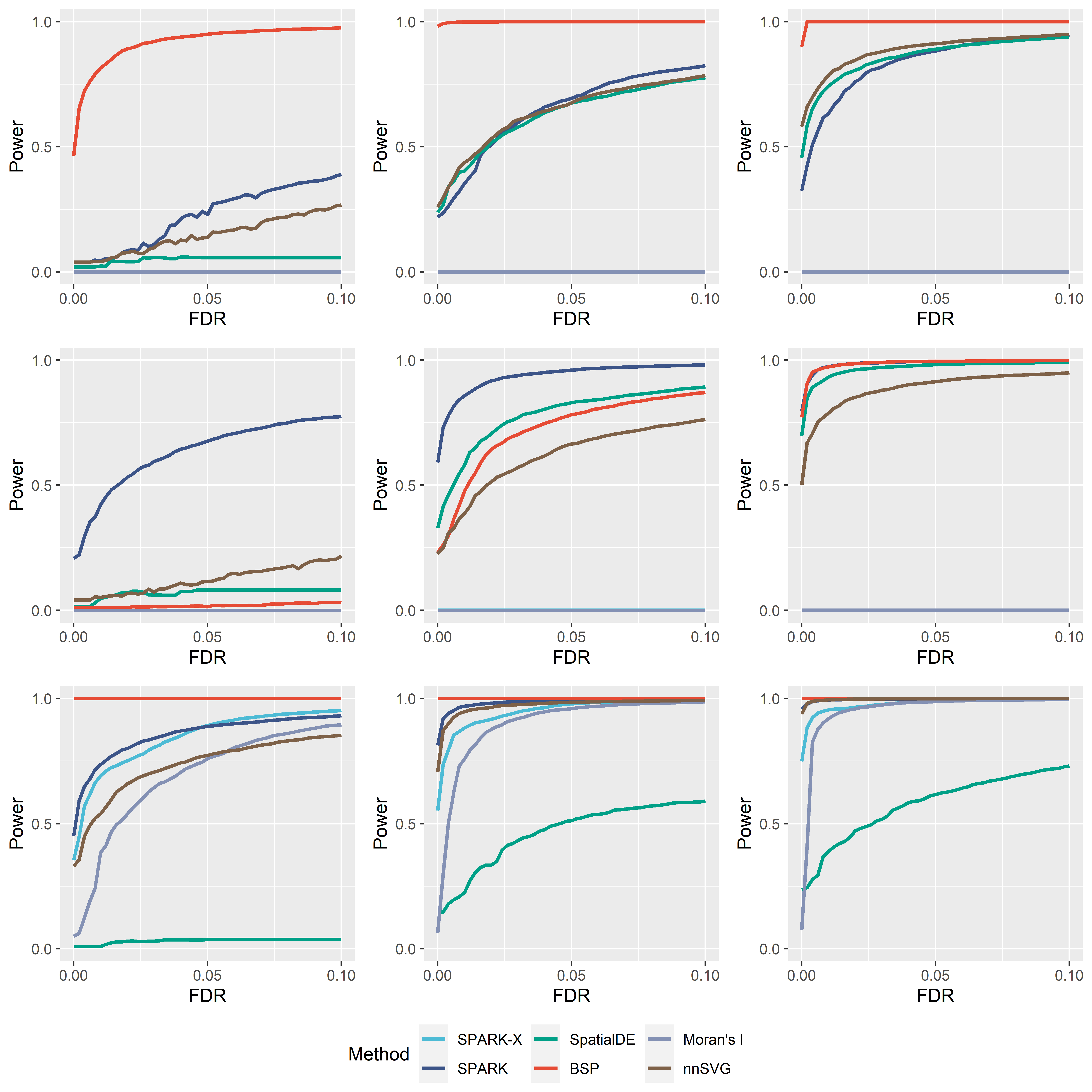


**Supplementary Figure 1. Power comparison of different methods with varying signal strengths in 2D simulations.** Power charts show the averaged true positive rates (y-axis) across ten replicates against the false discovery rates (x-axis) for the detected SVGs using each method. In these nine power charts, simulations with weak, moderate, and high signal strengths are shown in the left, middle, and right columns, respectively. Simulations using the spatial expression patterns I, II, and III are placed in the top, middle, and bottom rows, respectively. All simulated datasets were generated using a fixed moderate noise level.


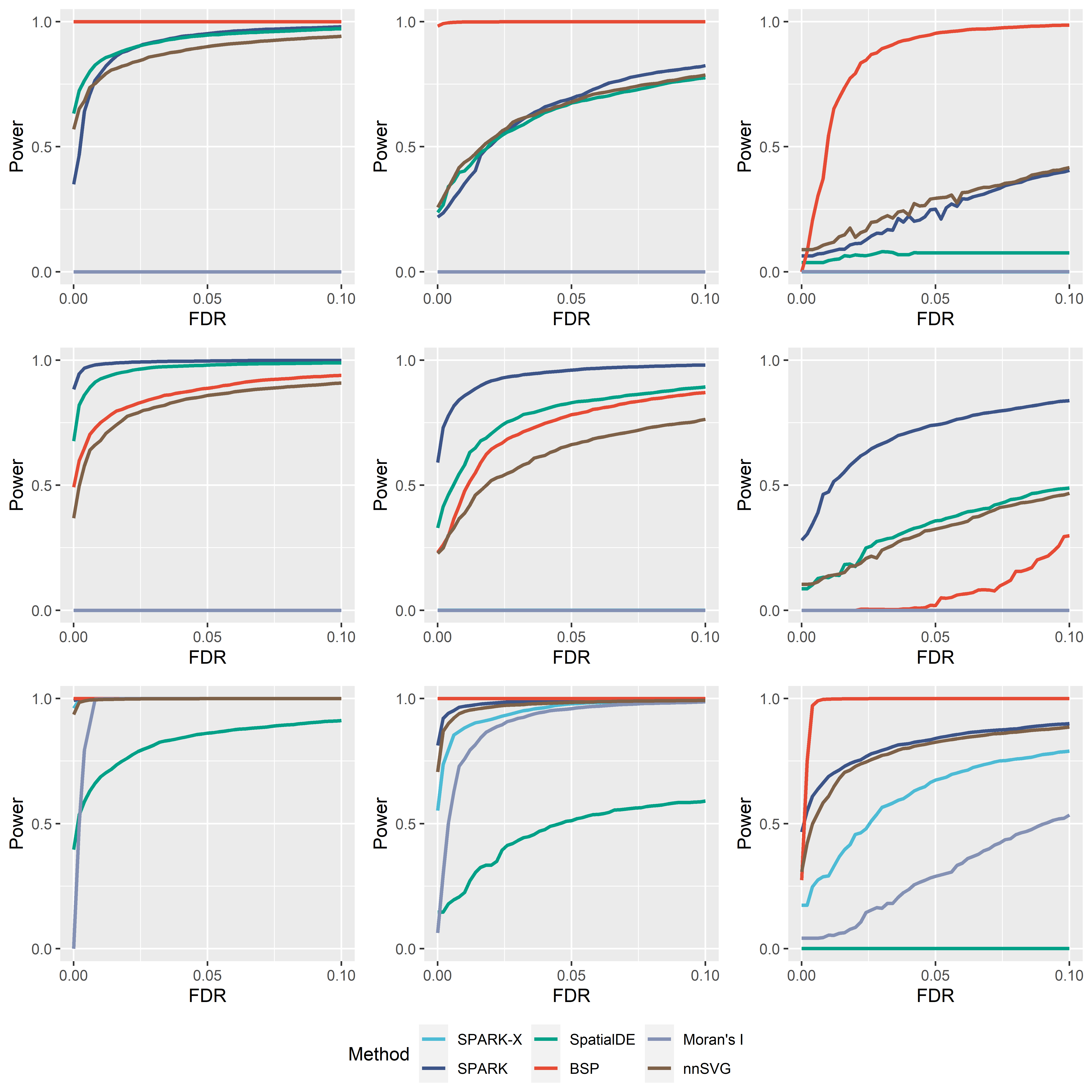


**Supplementary Figure 2: Power comparison of different methods with varying noise levels in 2D simulations.** Power charts show the averaged true positive rates (y-axis) across ten replicates against the false discovery rates (x-axis) for the detected SVGs using each method. These nine power charts show simulations with low, moderate, and high noise levels in the left, middle, and right columns, respectively. Simulations using the spatial expression patterns I, II, and III are placed in the top, middle, and bottom rows, respectively. All simulated datasets were generated using fixed moderate signal strength.


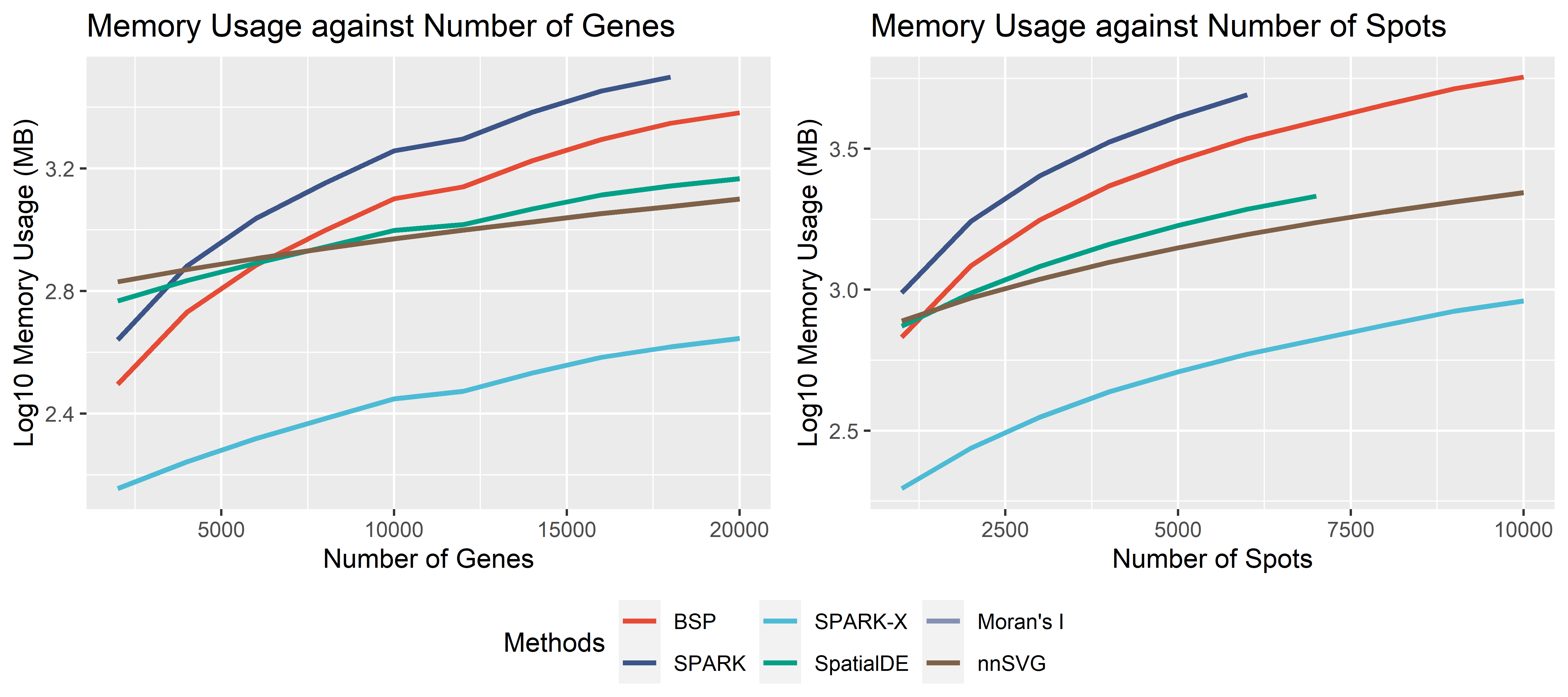


**Supplementary Figure 3. BSP memory usage with different gene numbers and different numbers of spots.** The experiments are performed on an Ubuntu 16.04.4 LTS workstation with Intel(R) Xeon(R) W-2125 CPU @ 4.00GHz and 32 GB memory.

**
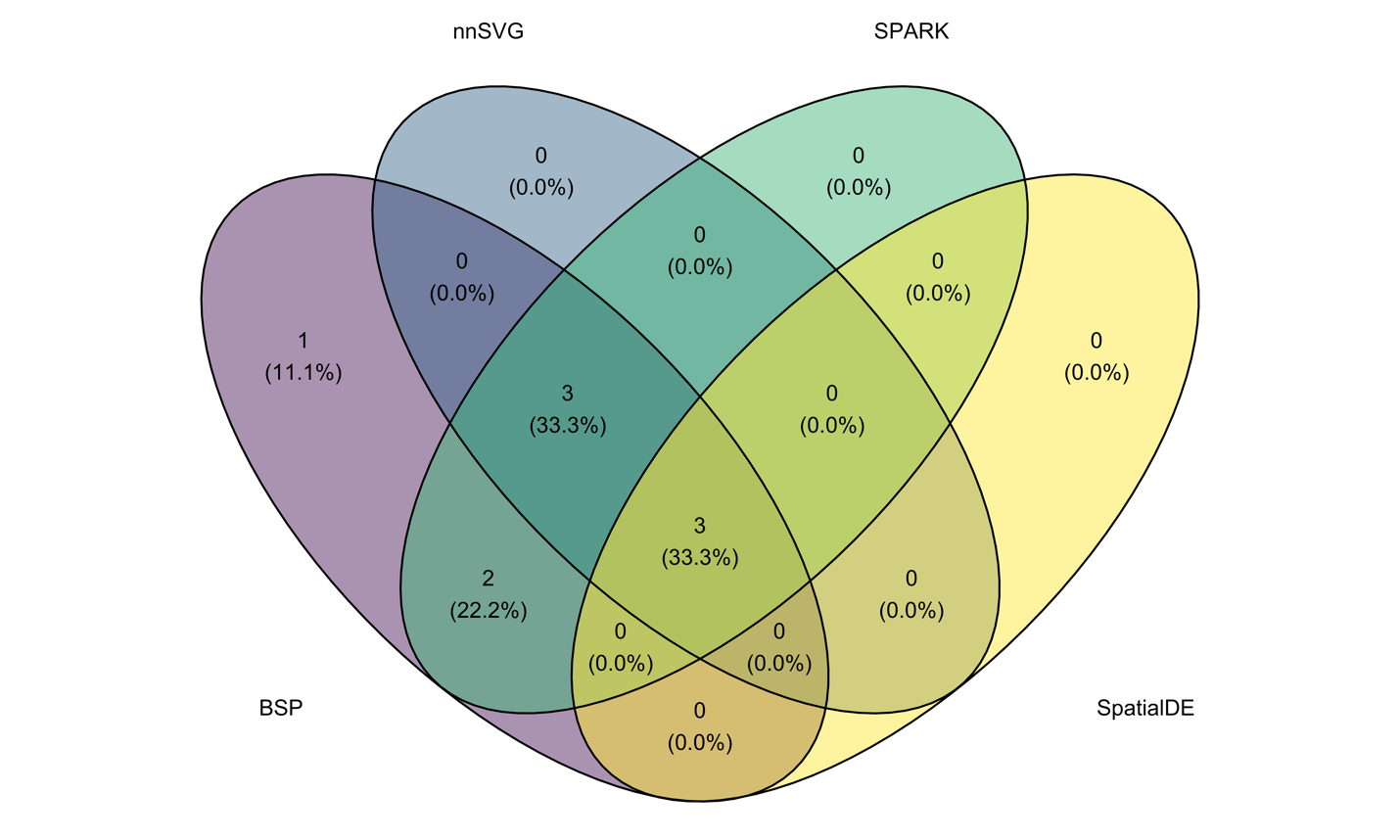
**

**Supplementary Figure 4. Venn diagram of marker genes identified by BSP, nnSVG, SPARK, and SpatialDE in mouse olfactory bulb research**. The original study includes 10 marker genes.

**
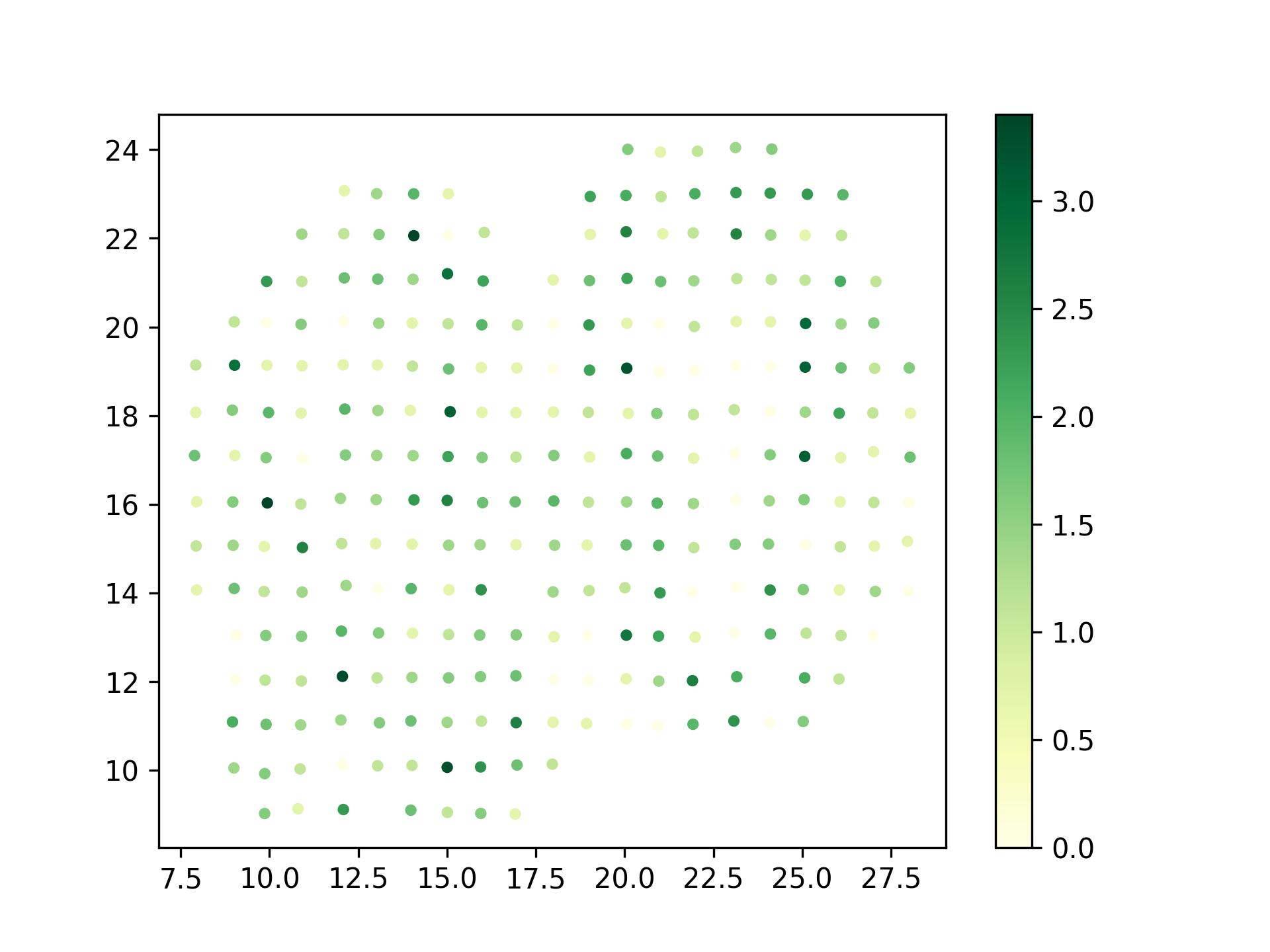
**

**Supplementary Figure 5. Missed marker gene** *Sv2b* **by BSP in mouse olfactory bulb study**. Colors indicate gene expression levels. The expression values were log-transformed, and those greater than 1.0 were normalized to 1.0.

**
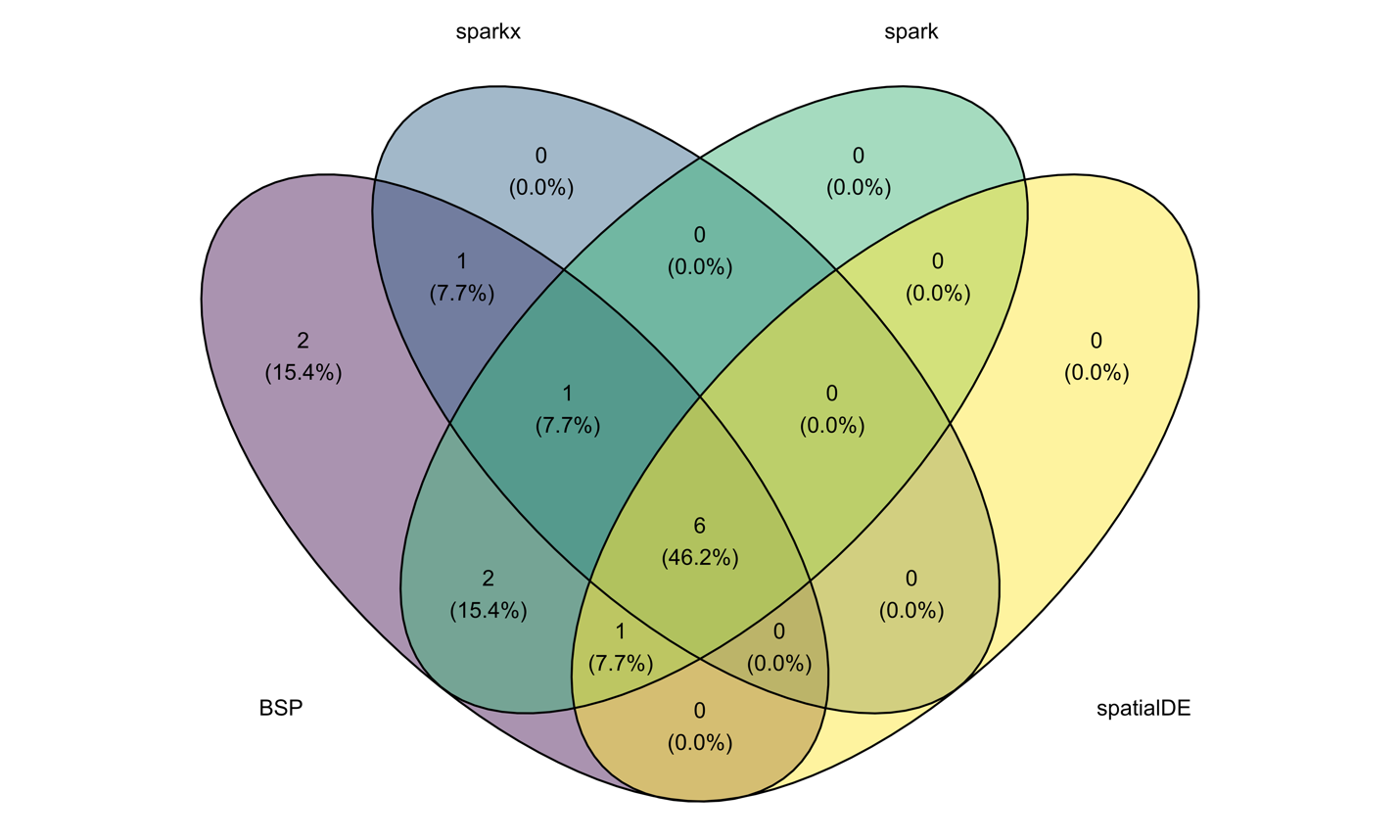
**

**Supplementary Figure 6. Venn diagram of marker genes identified by BSP, SPARK, SPARKX, and SpatialDE in human breast cancer research**. The original study identified 14 marker genes.


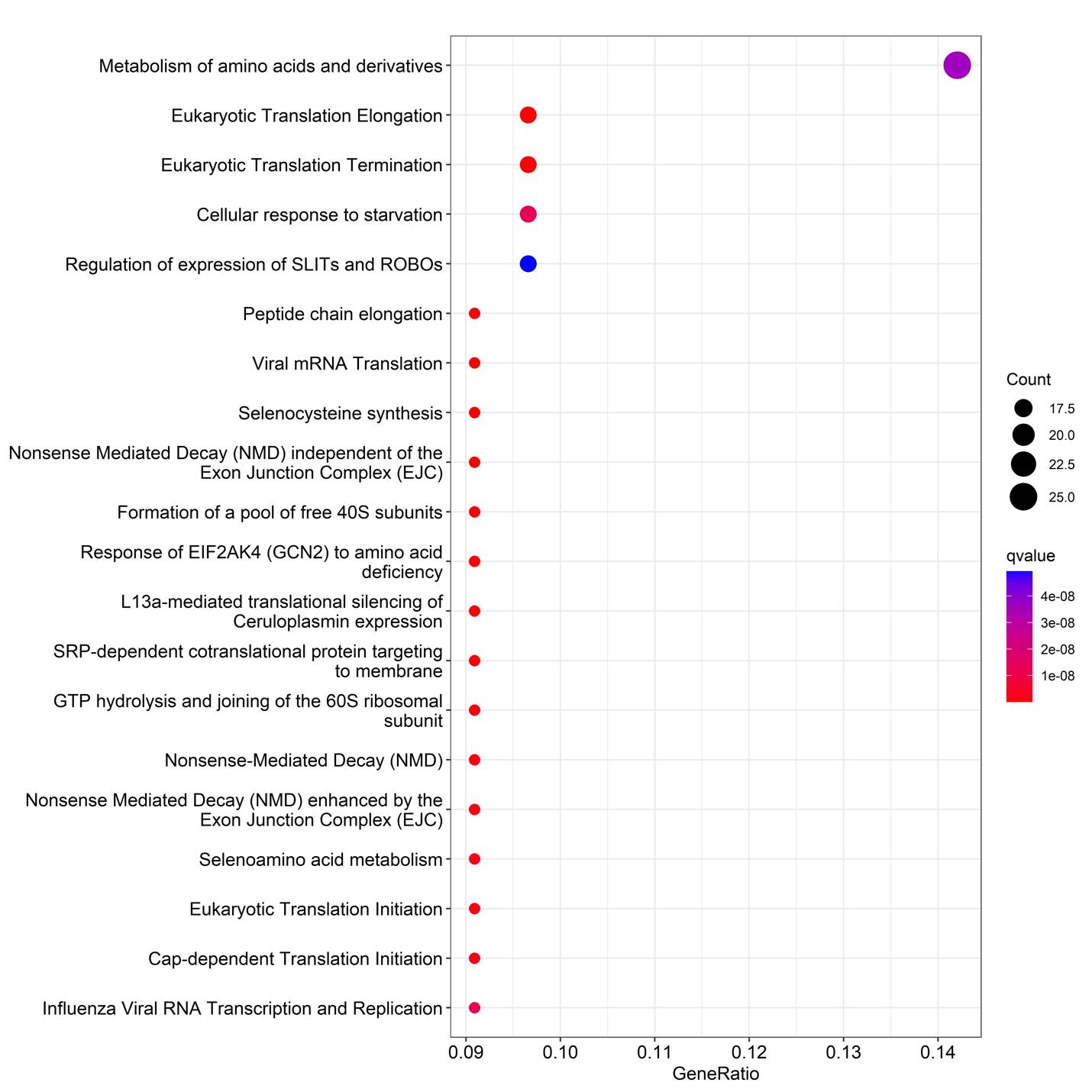


**Supplementary Figure 7: Pathway enrichment analysis on SVGs in AKI study using 10X Visium.** P-values are one-sided Fisher's exact test adjusted by FDR.

**
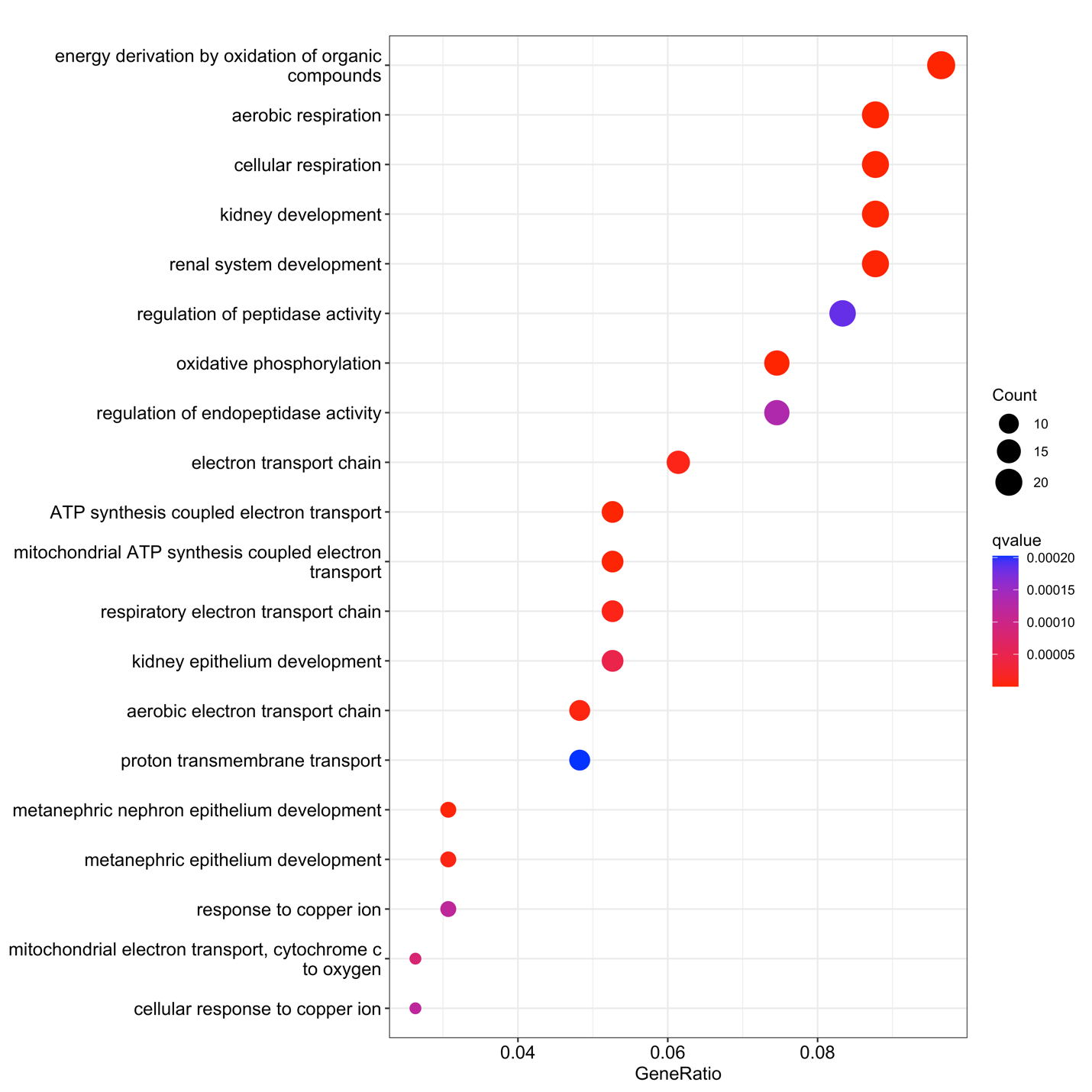
**

**Supplementary Figure 8: GO enrichment analysis on SVGs of Pattern 1 in AKI study using 10X Visium.** P-values are one-sided Fisher's exact test adjusted by FDR.

**
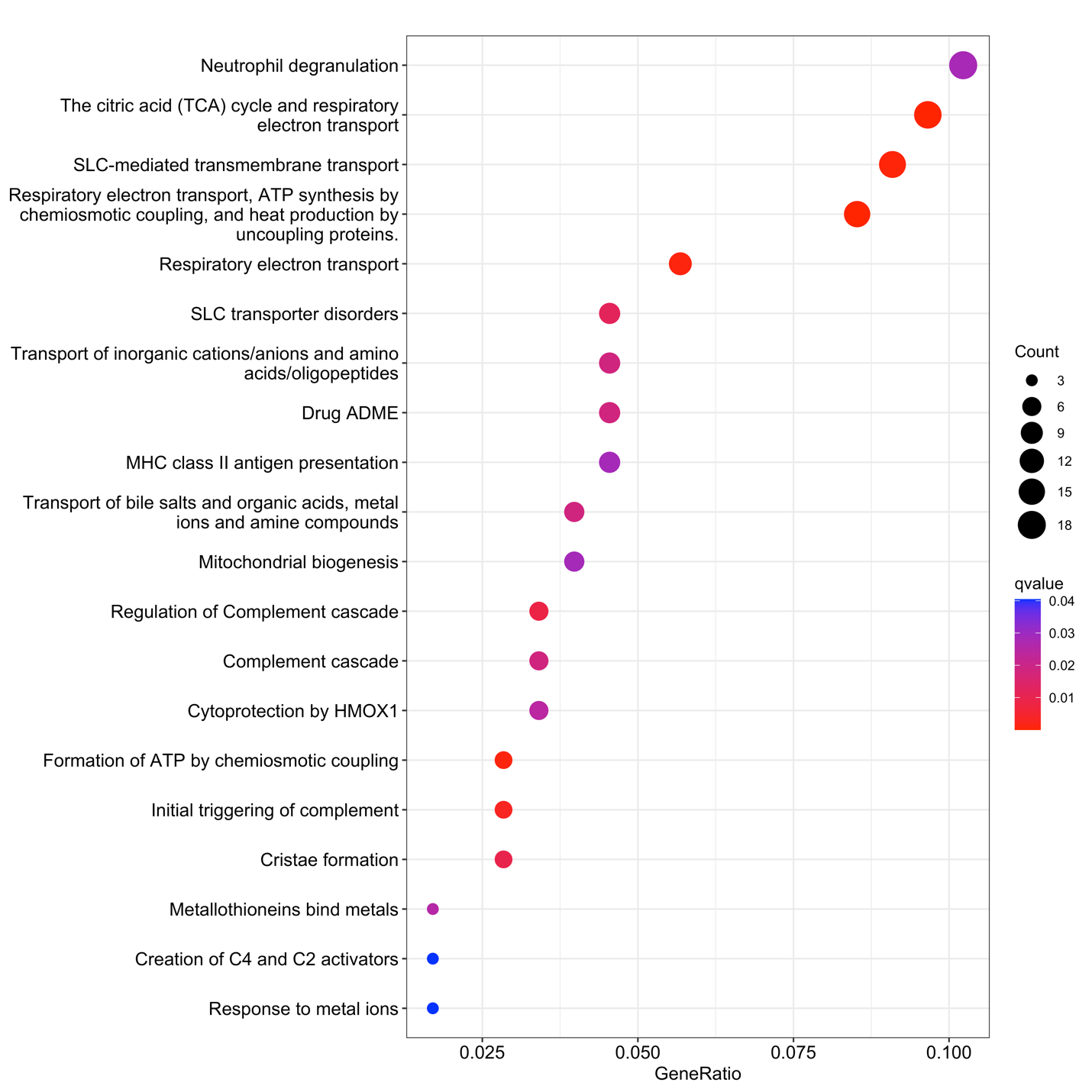
Supplementary Figure 9: Pathway enrichment analysis on SVGs of Pattern 1 in AKI study using 10X Visium.** P-values are one-sided Fisher's exact test adjusted by FDR.

**
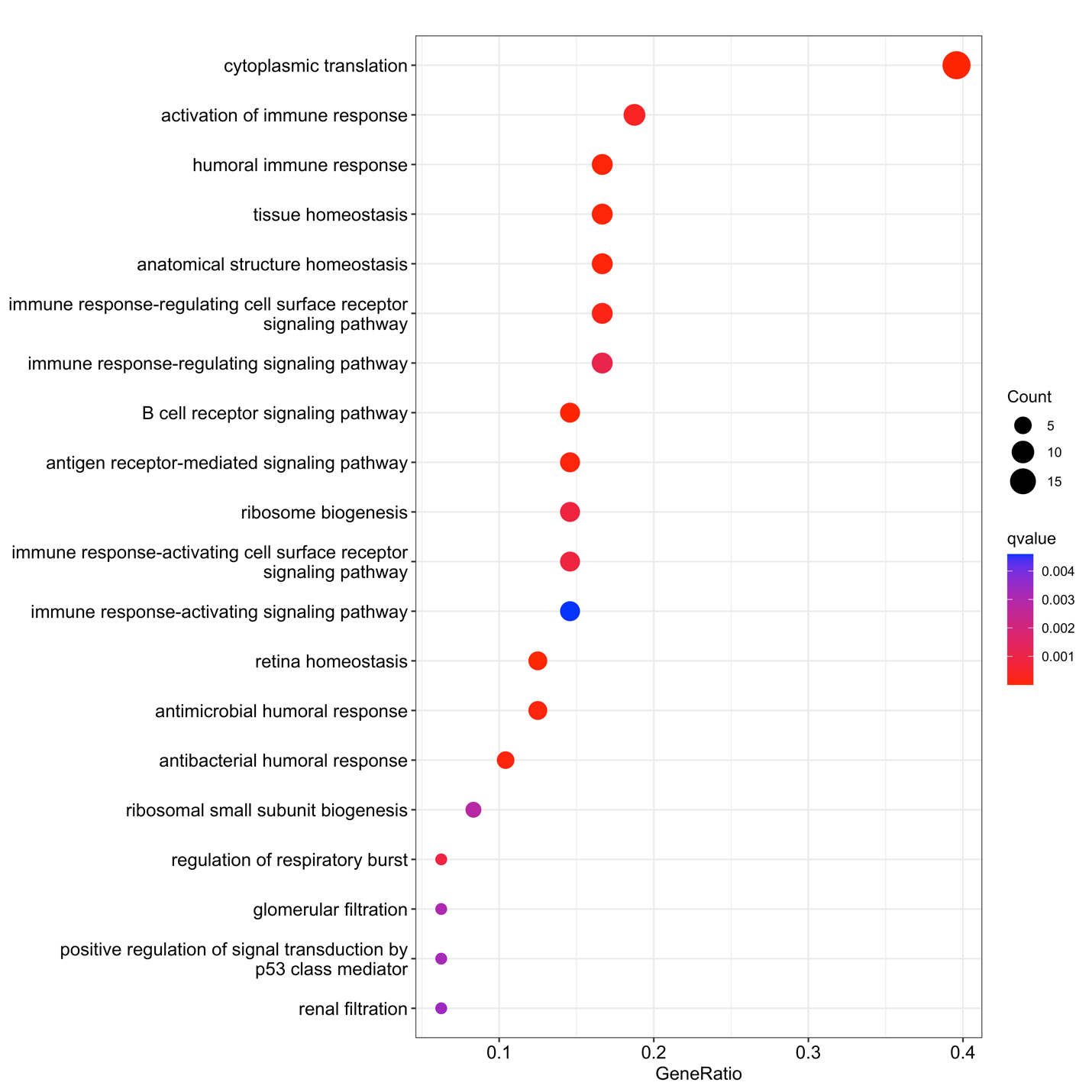
Supplementary Figure 10: GO enrichment analysis on SVGs of Pattern 2 in AKI study using 10X Visium.** P-values are one-sided Fisher's exact test adjusted by FDR.

**
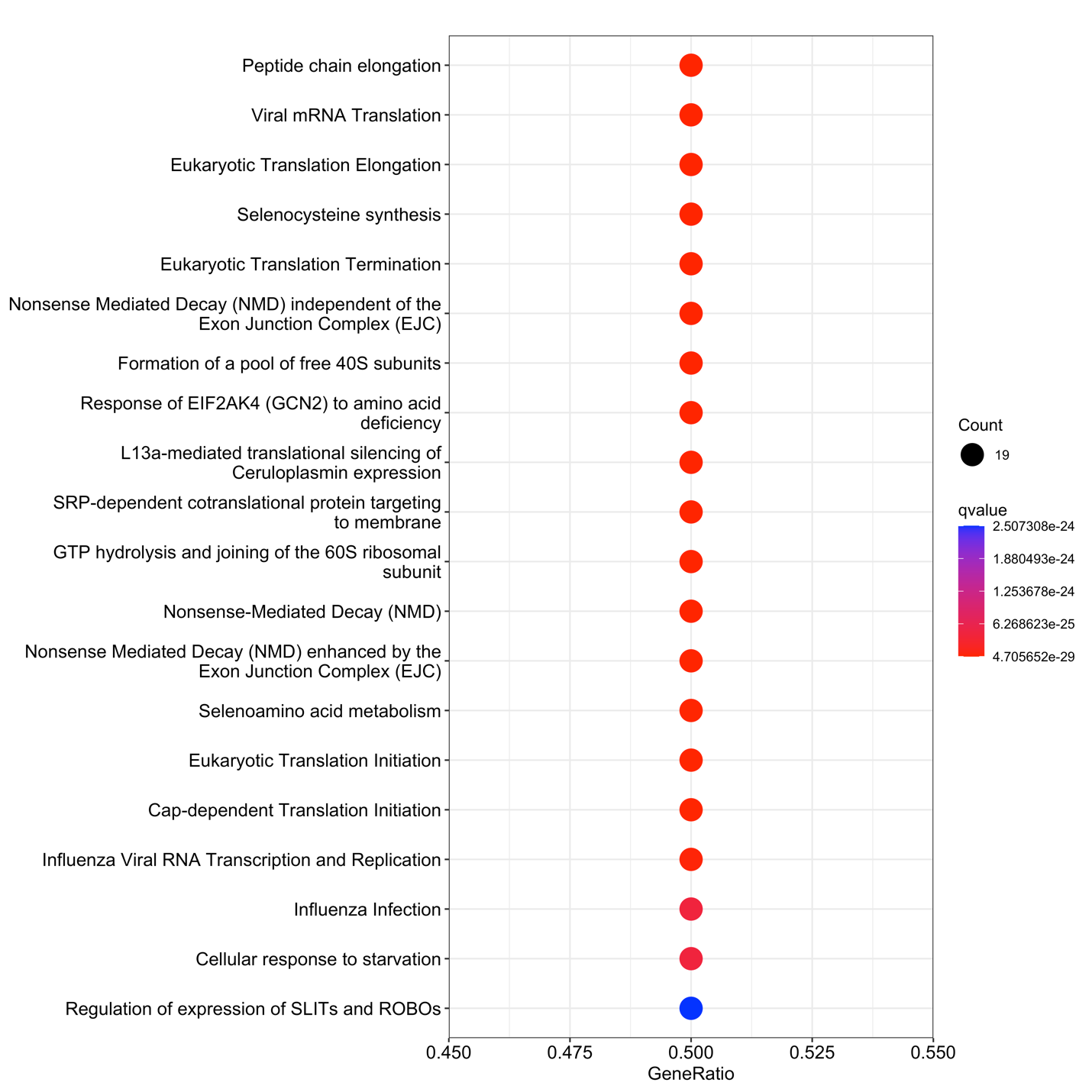
Supplementary Figure 11: Pathway enrichment analysis on SVGs of Pattern 2 in AKI study using 10X Visium.** P-values are one-sided Fisher's exact test adjusted by FDR.


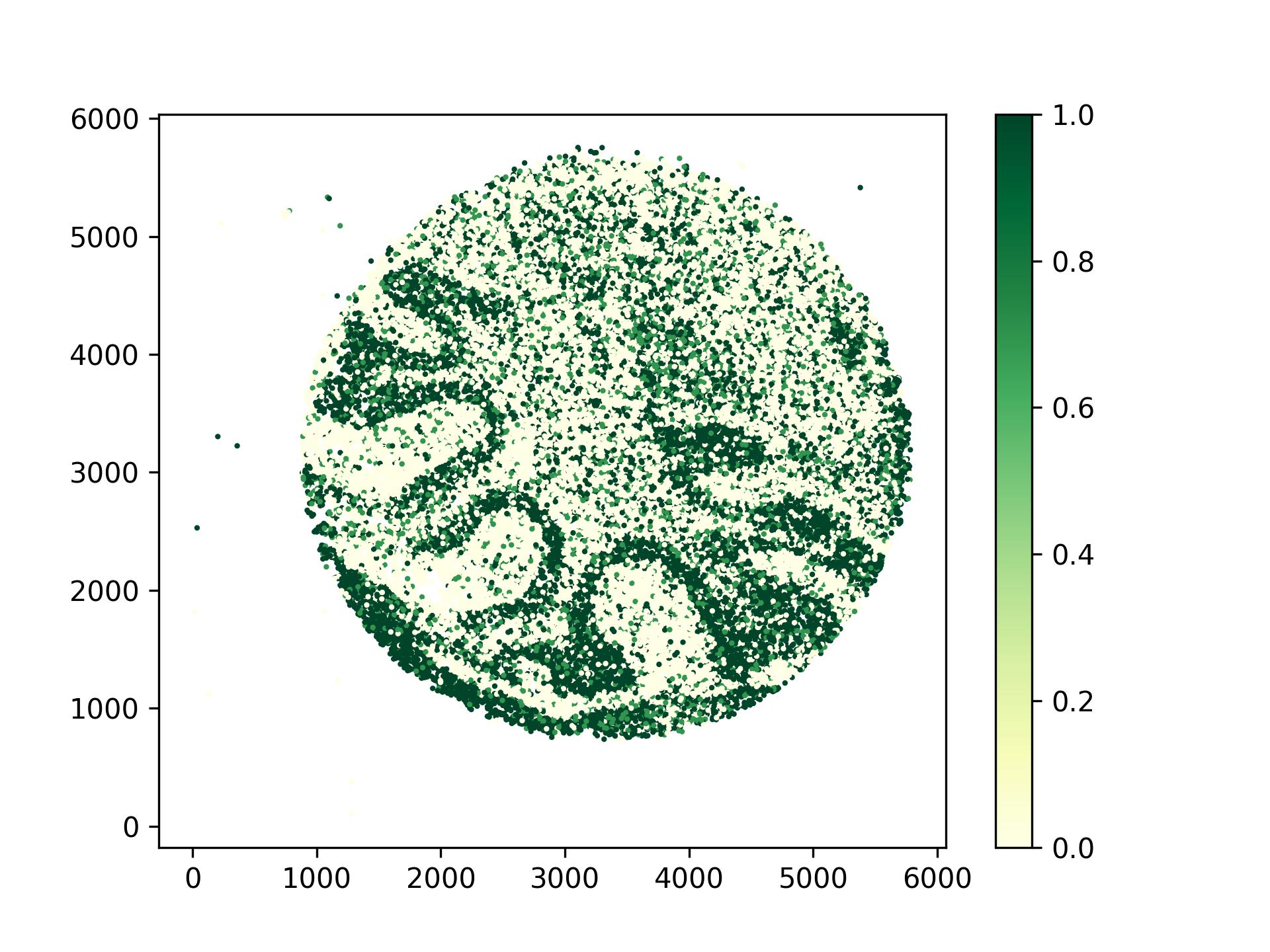


**Supplementary Figure 12. Showcase of** *Malat1* **as an SVG identified by BSP.** Allen Brain Atlas does not have expression or ISH available at mouse.brain-map.org. Colors indicate gene expression levels. The expression values were log-transformed, and those greater than 1.0 were normalized to 1.0.


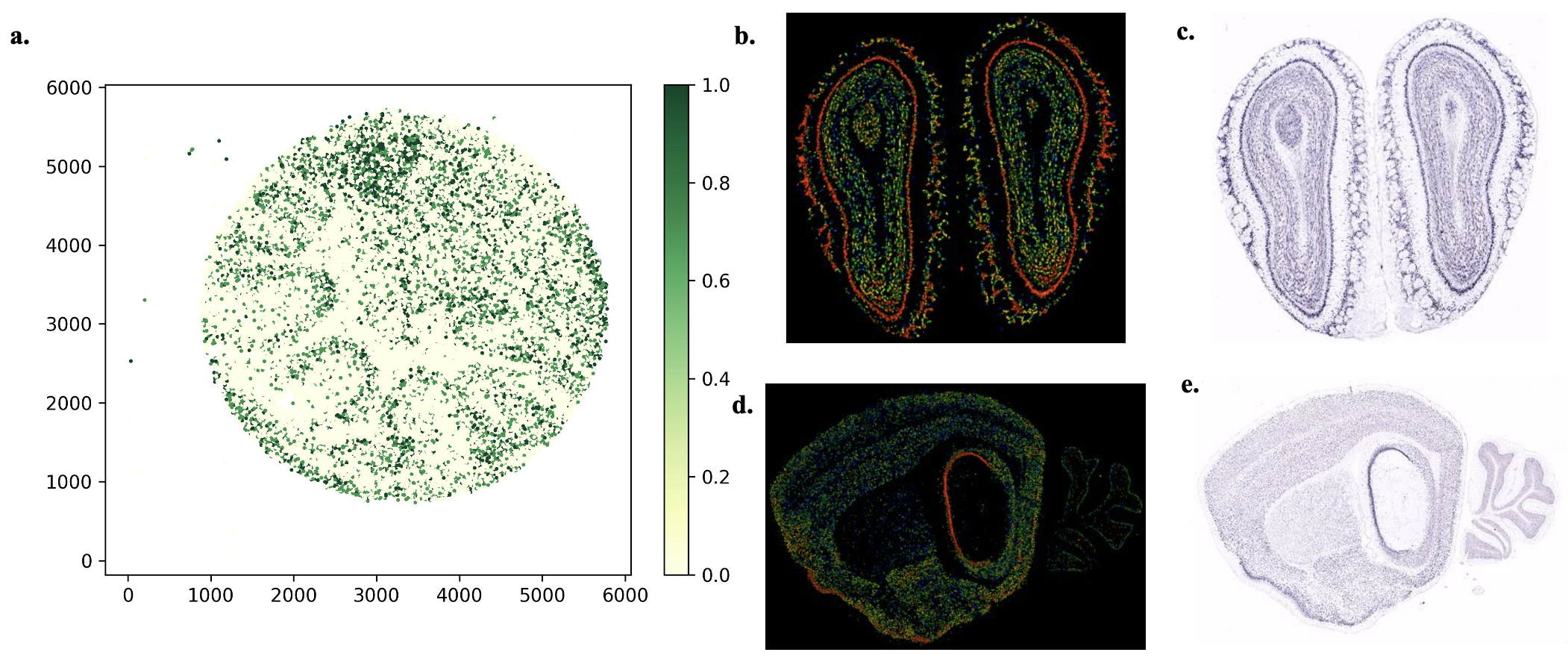


**Supplementary Figure 13. Showcase of** *Ttc3* **as an SVG identified by BSP.** **a)** *Ttc3* gene expression in the mouse cerebellum data using Slide-seq V2; **b)** Expression and **c)** ISH of *Ttc3* gene in coronal adult mouse brain <http://mouse.brain-map.org/experiment/show/1079>; **d)** Expression and **e)** ISH of *Ttc3* gene in an sagittal adult mouse brain <http://mouse.brain-map.org/experiment/show/1080>.

**
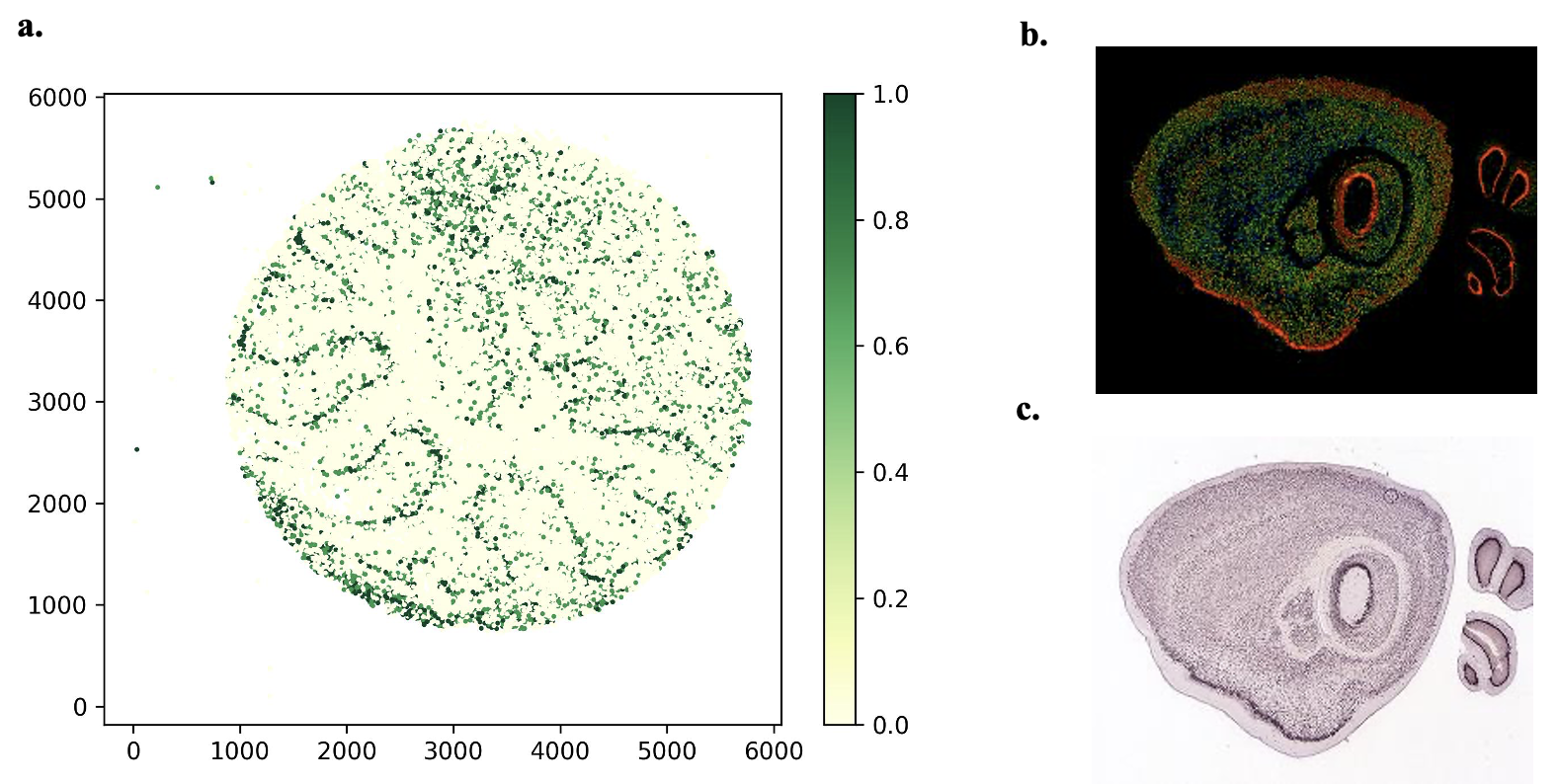
**

**Supplementary Figure 14. Showcase of** *Nsg1* **as an SVG identified by BSP**. **a)** *Nsg1* gene expression in the mouse cerebellum data using Slide-seq V2; **b)** Expression and **c)** ISH of *Nsg1* gene in sagittal adult mouse brain <http://mouse.brain-map.org/experiment/show/70429327>.

**
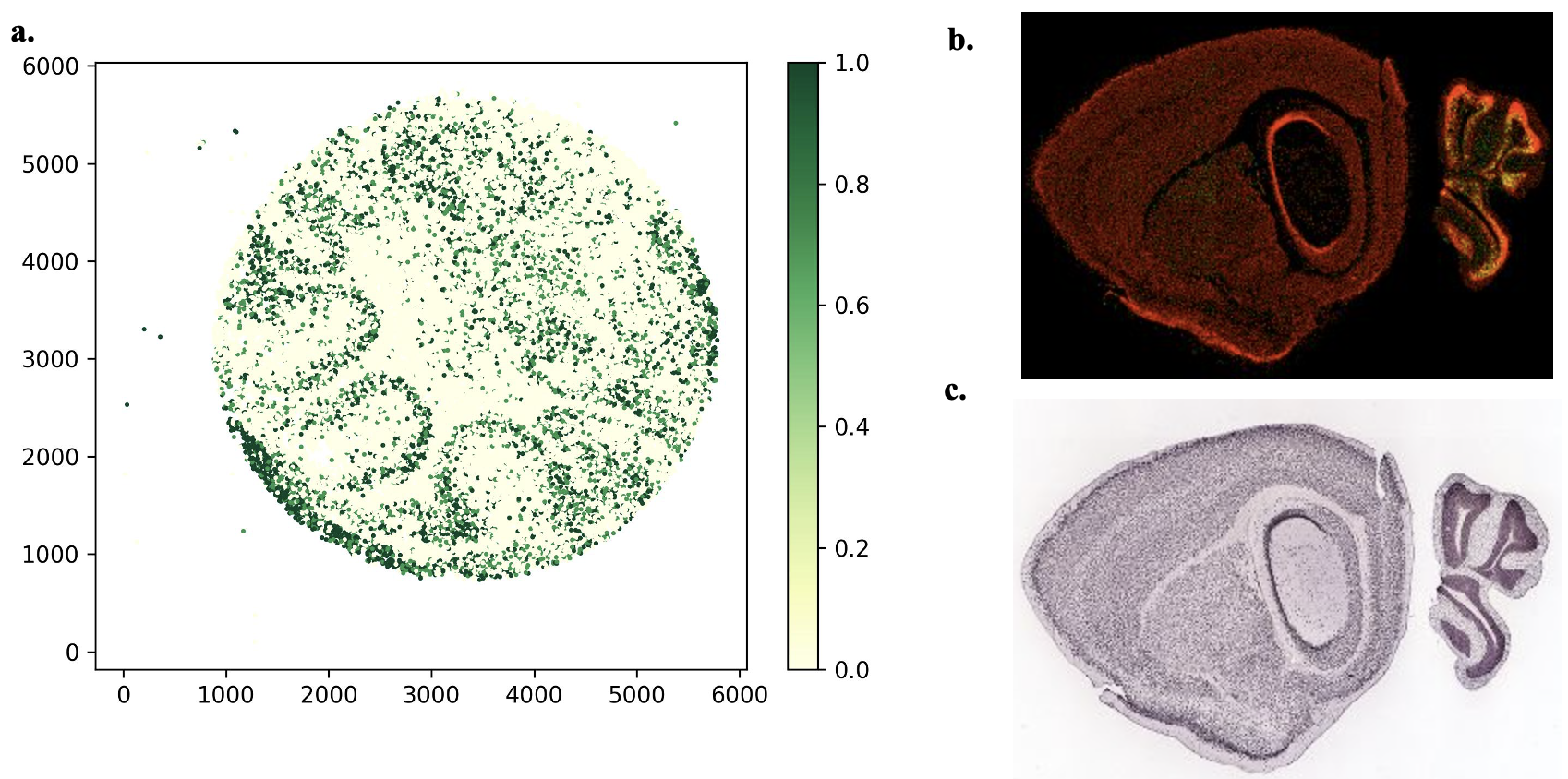
 Supplementary Figure 15. Showcase of** *Meg3* **as an SVG identified by BSP. a)** *Meg3* gene expression in the mouse cerebellum data using Slide-seq V2; **b)** Expression and **c)** ISH of *Sparcl1* gene in sagittal adult mouse brain <http://mouse.brain-map.org/experiment/show/71281027>.


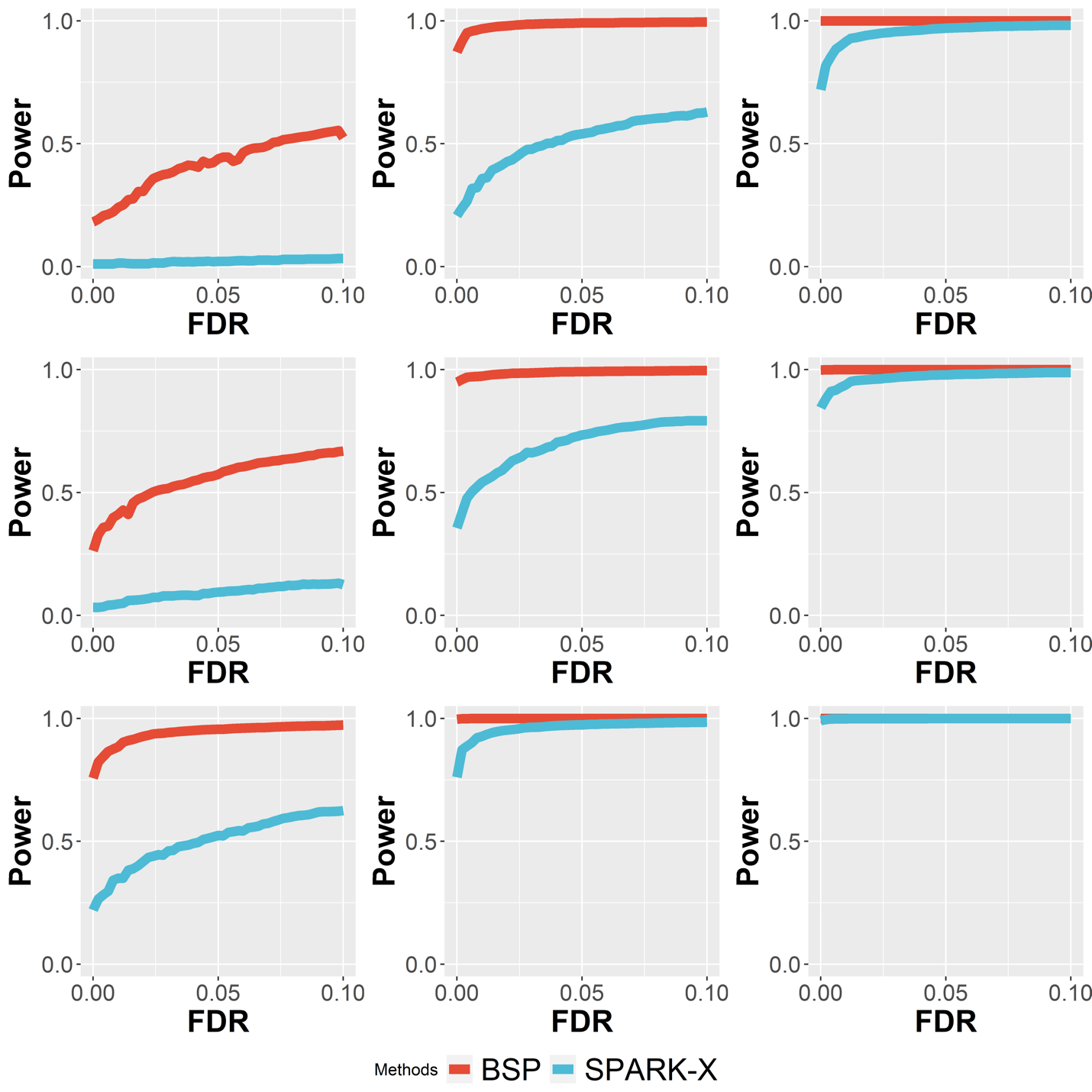


**Supplementary Figure 16: Power comparison with varying pattern sizes in 3D simulations**. Simulations were performed using a fixed moderate signal strength and low noise level. In these nine power charts, simulations with small, moderate, and large pattern sizes are in the left, middle, and right columns, respectively. Simulations using the continuous 3D Pattern I (curved stick), Pattern II (thin plate), and Pattern III (irregular lump) are in the top, middle, and bottom rows, respectively.


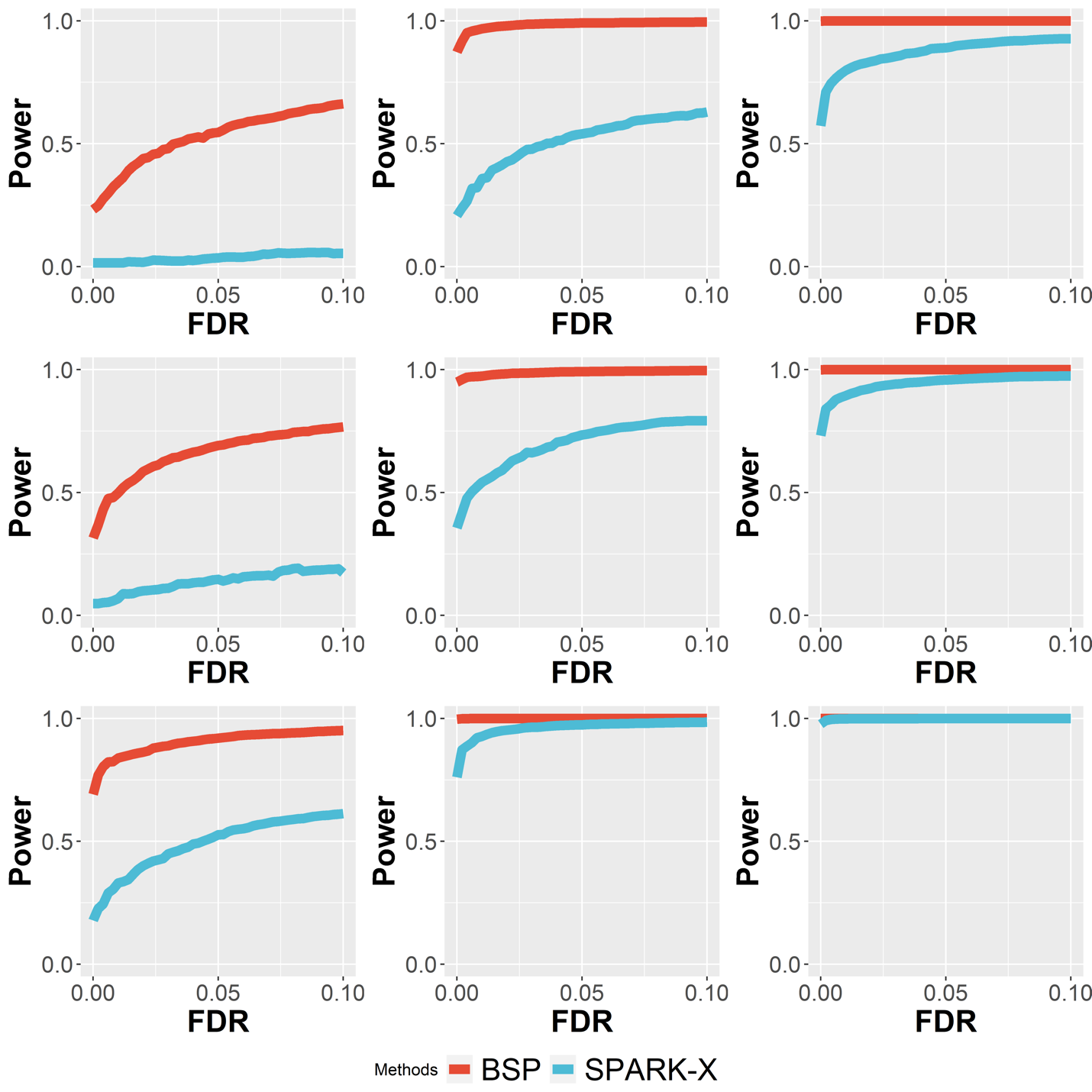


**Supplementary Figure 17: Power comparison with varying signal strengths in 3D simulations.** Simulations were performed using a fixed moderate pattern size and low noise level. In these nine power charts, simulations with signal strengths as low, moderate, and high are in the left, middle, and right columns, respectively. Simulations using the continuous 3D Pattern I (curved stick), pattern II (thin plate), and Pattern III (irregular lump) are in the top, middle, and bottom rows, respectively.


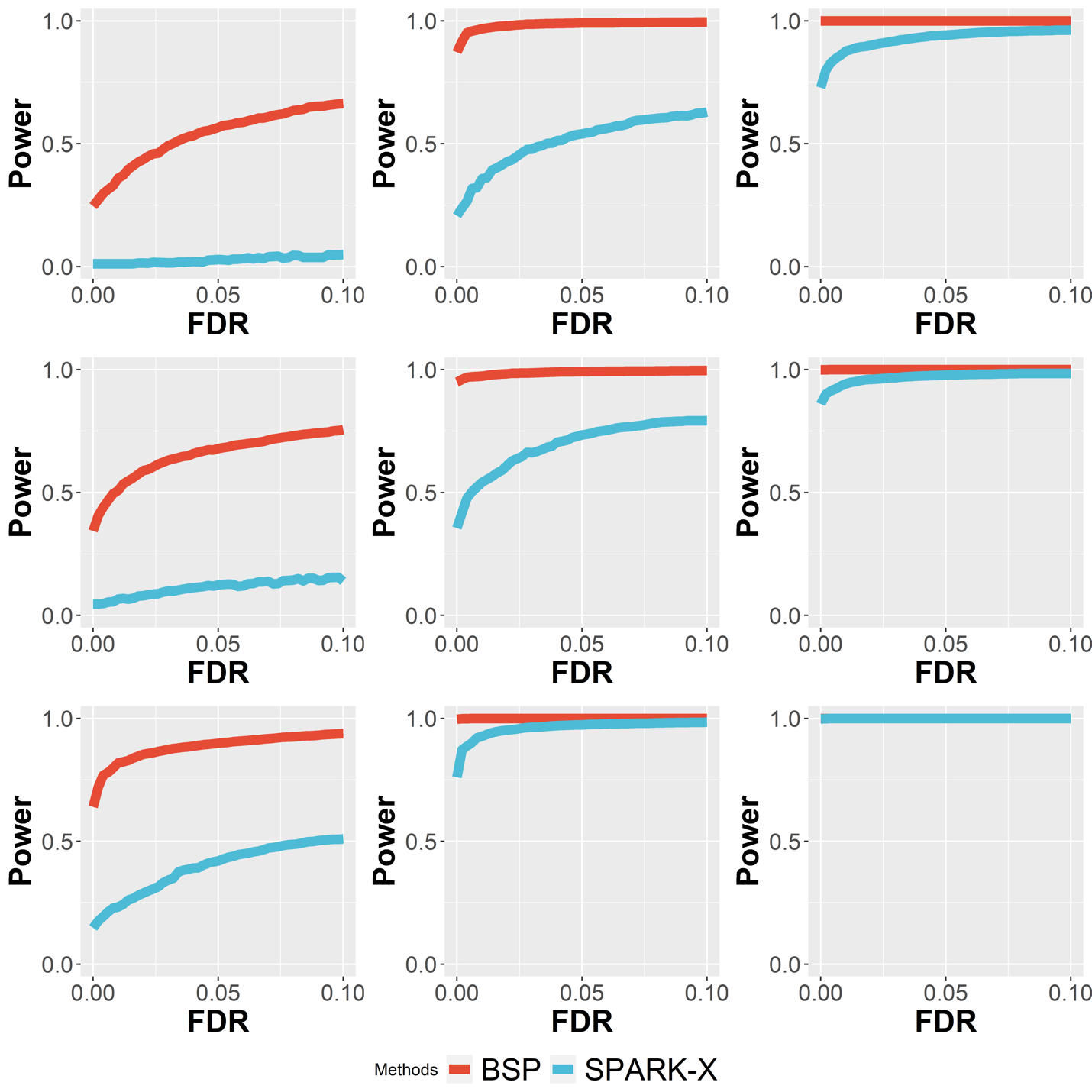


**Supplementary Figure 18: Power comparison with varying noise levels in 3D simulations.** Simulations were performed using a fixed moderate pattern size and moderate signal strength. In these nine power charts, simulations with high, moderate, and low noise levels are in the left, middle, and right columns, respectively. Simulations using the 3D Pattern I (curved stick), pattern II (thin plate), and Pattern III (irregular lump) are shown in the top, middle, and bottom rows, respectively.


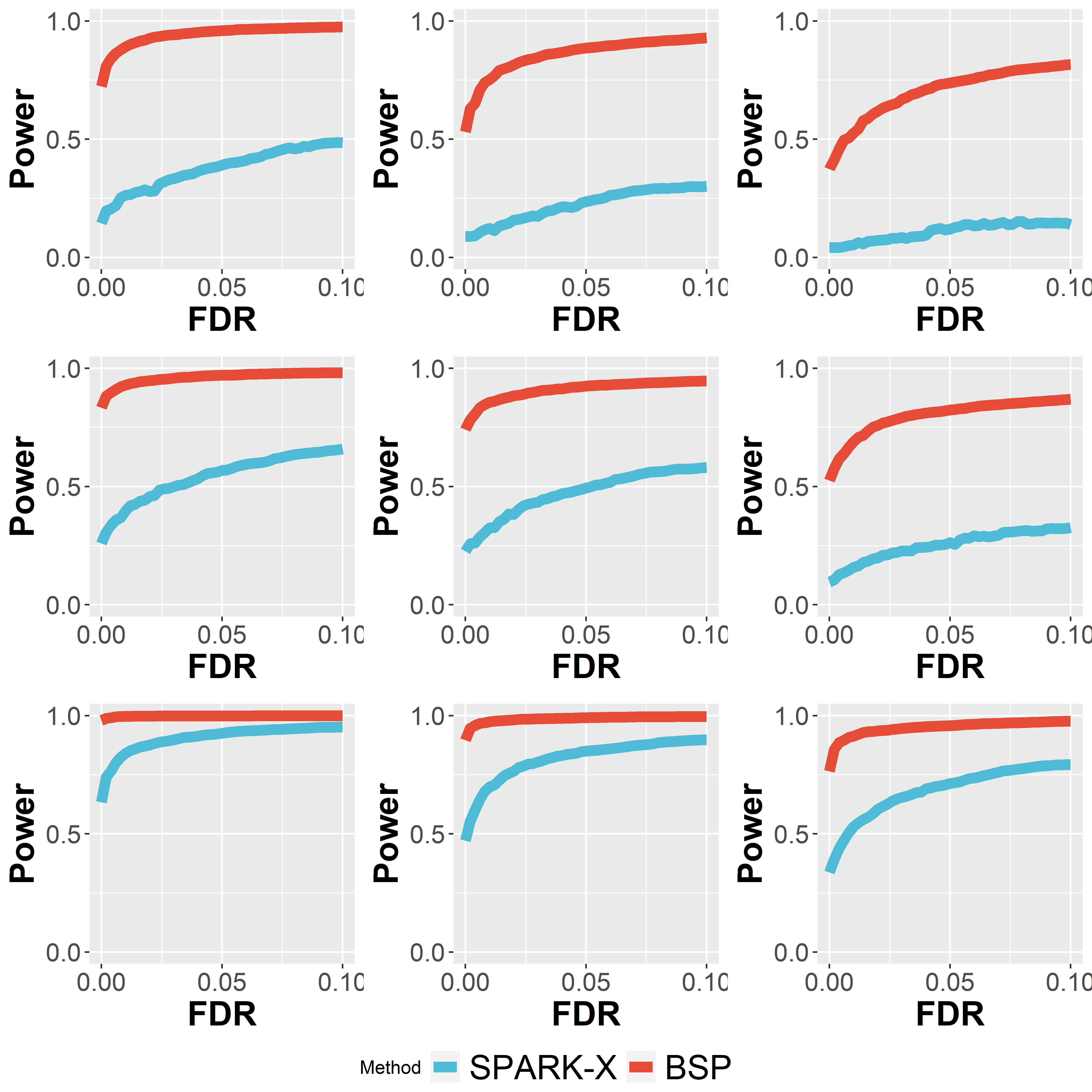


**Supplementary Figure 19: Power comparisons with varying dropout rates.** Simulations were performed using a fixed moderate pattern size, moderate signal strength, and moderate noise level. In these nine power charts, simulations with low (10%), moderate (20%), and high (30%) dropout rates are in the left, middle, and right columns, respectively. Simulations using the continuous 3D Pattern I (curved stick), Pattern II (thin plate), and Pattern III (irregular lump) are shown in the top, middle, and bottom rows, respectively.


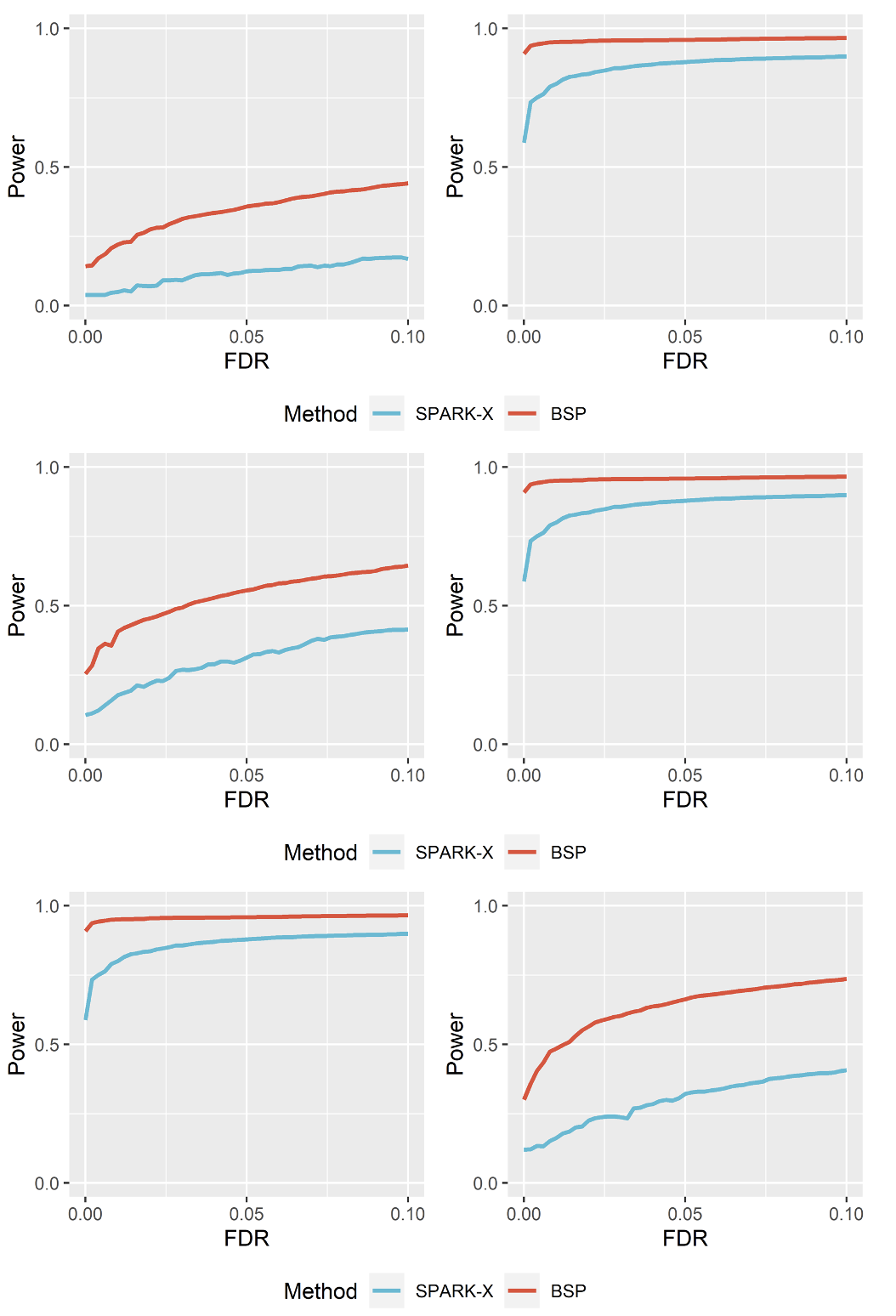


**Supplementary Figure 20: Performances on discrete and continuous spatial patterns.** This simulation contains 500 SVGs with discrete patterns, 500 SVGs with continuous patterns, and 9,000 non-SVGs. Simulations were conducted with varied pattern sizes (small to moderate, left to right), signal strength (small to moderate, left to right), and noise level (moderate to high, left to right) while holding the rest two parameters (top to bottom).


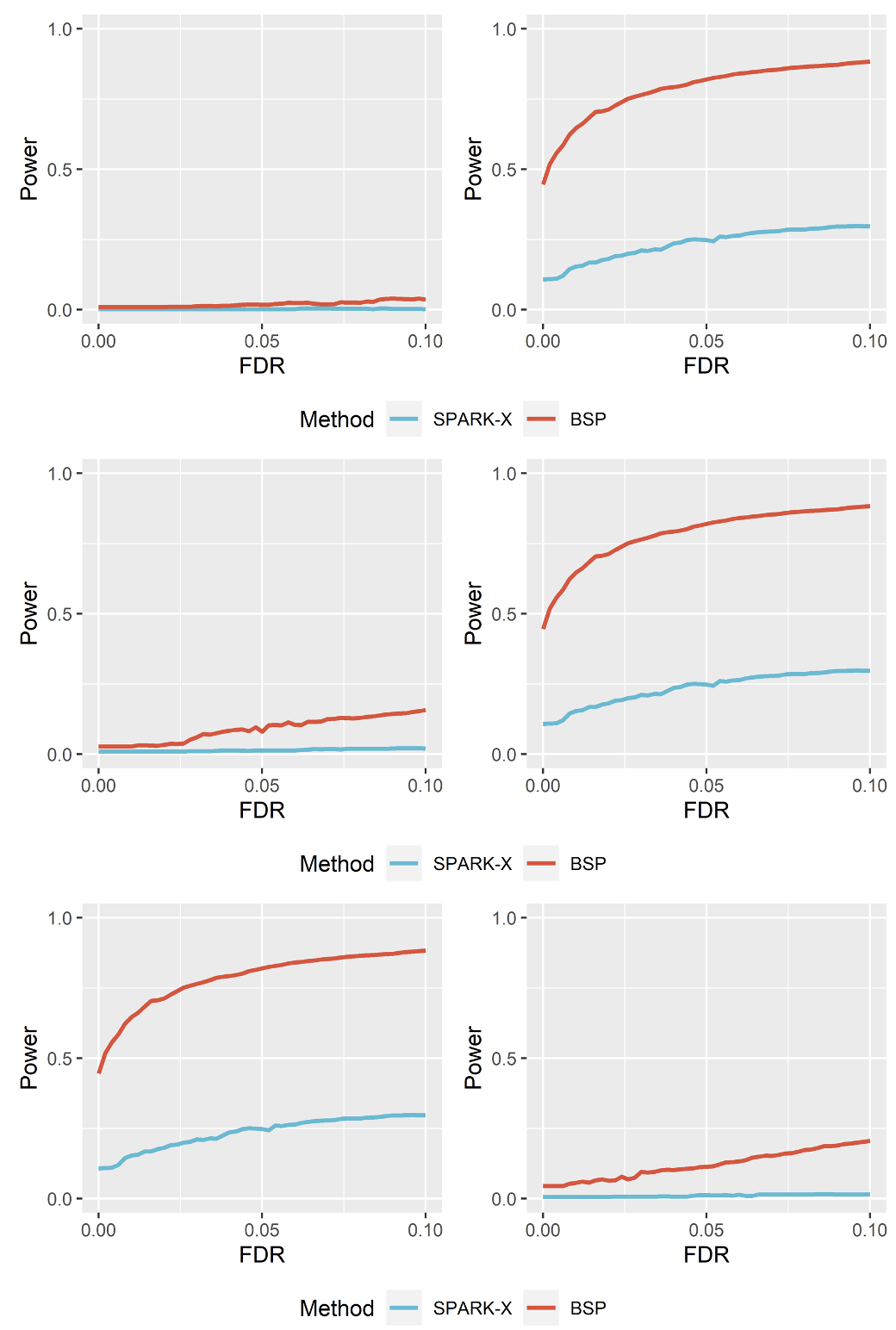


**Supplementary Figure 21: Performances on 3D simulations within inconsistent within-plane and inter-plane resolution.** Simulations were conducted with varied pattern sizes (small to moderate, left to right), signal strength (low to moderate, left to right), and noise level (moderate to high, left to right) while holding the rest two parameters as moderate (top to bottom).

**
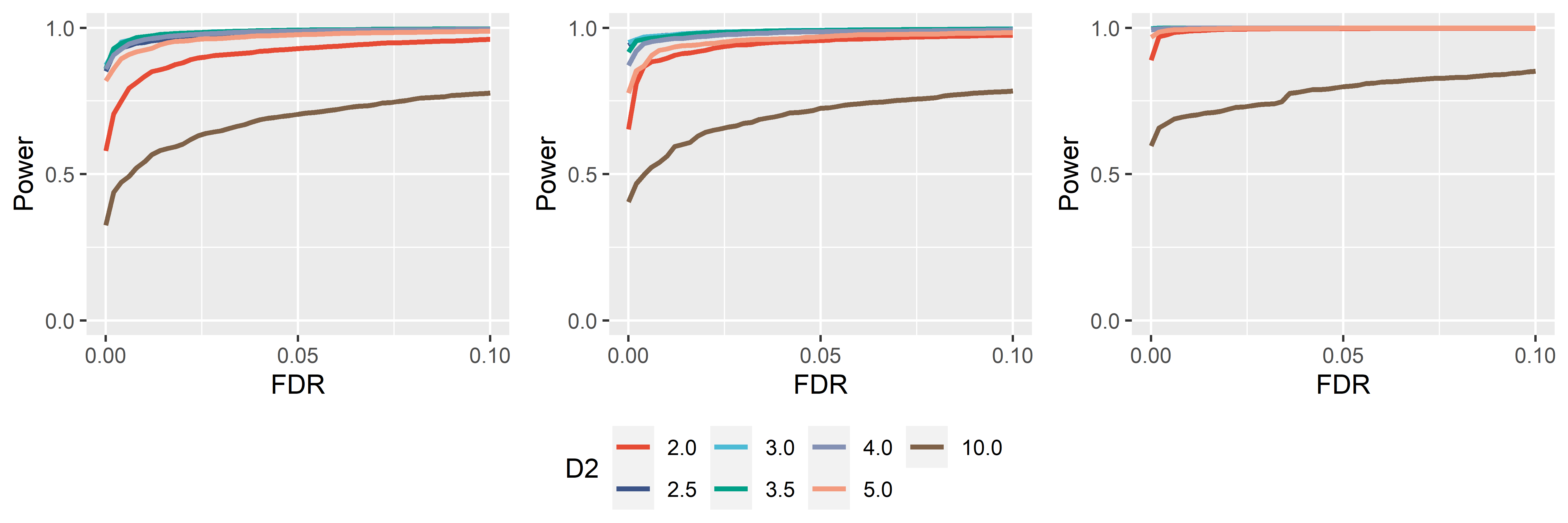
**

**Supplementary Figure 22: Power comparisons with gradient scales of big-small-batch.** The power analysis was conducted for a series of D2 values in the BSP model. Simulations using the continuous 3D pattern I (curved stick), pattern II (thin plate), and pattern III (irregular lump) are shown in the left, middle, and right columns, respectively. The results show very slight differences when D2 ranges from 2.0 to 5.0.

**
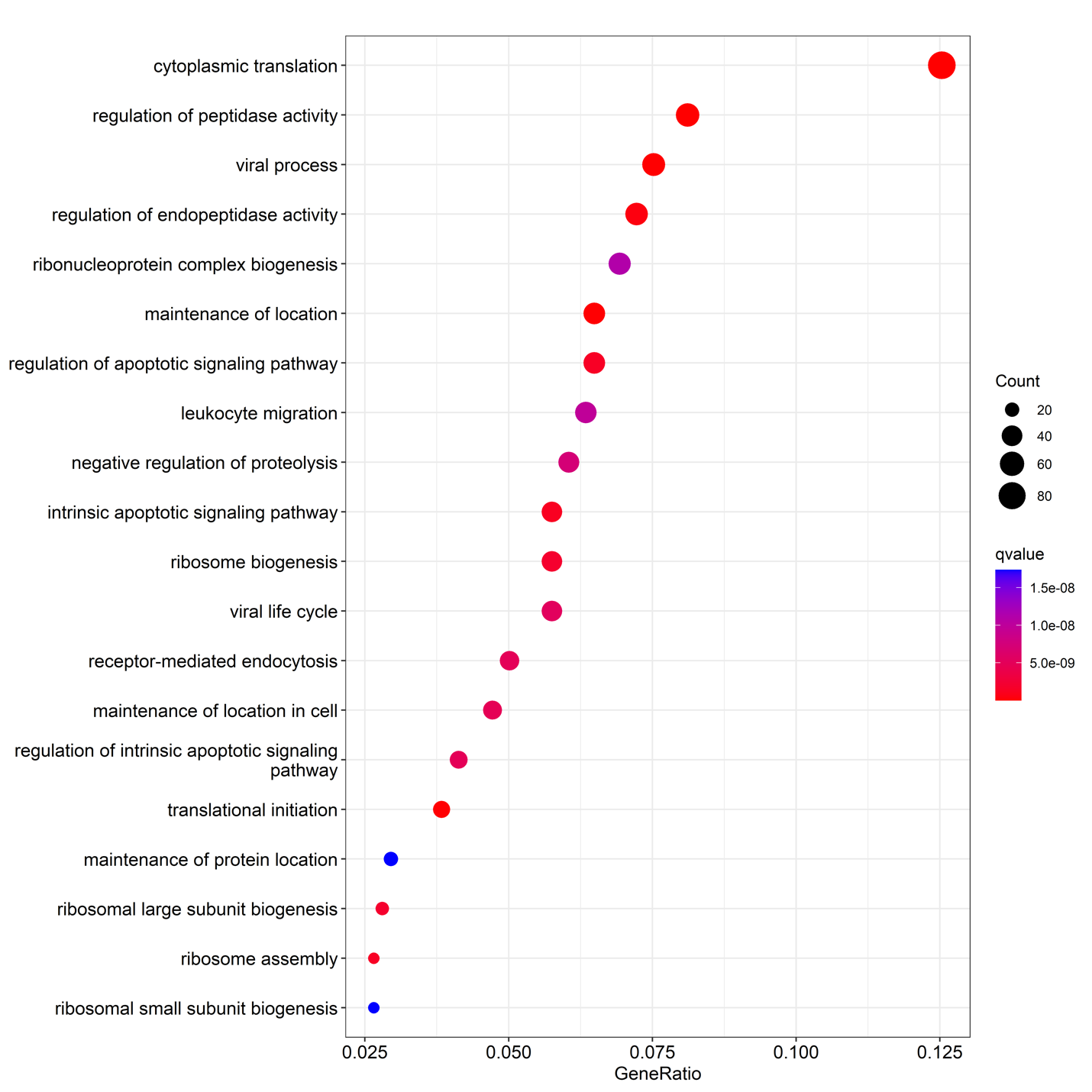
**

**Supplementary Figure 23: Gene ontology enrichment analysis on 724 genes both identified by 2D meta-analysis and 3D settings in patient RA1.** P-values are one-sided Fisher's exact test adjusted by FDR.


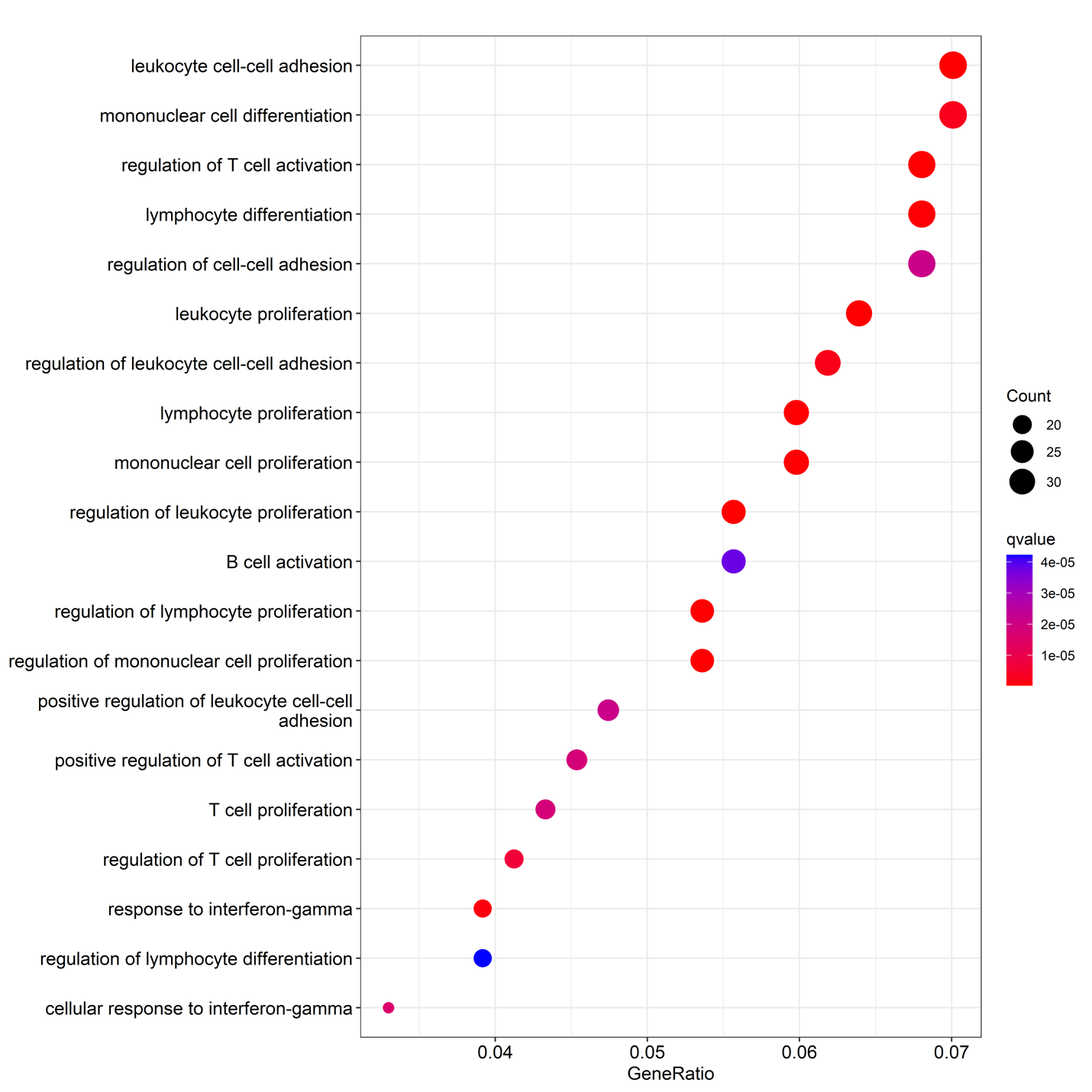


**Supplementary Figure 24: Gene ontology enrichment analysis on 532 genes uniquely identified by 3D settings in patient RA1.** P-values are one-sided Fisher's exact test adjusted by FDR.


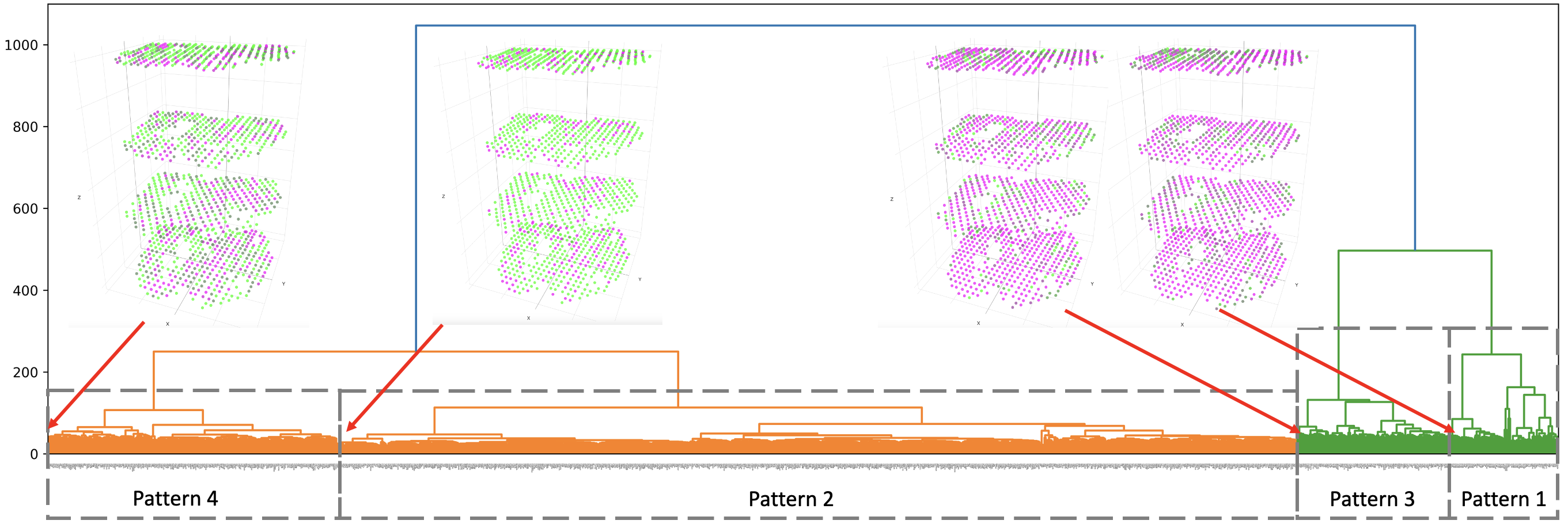


**Supplementary Figure 25: Hierarchical clustering identified four spatial patterns of SVGs of the RA1 sample using 3D SRT.** From left to right: Pattern 4, represented by gene *ACP5*; Pattern 2, represented by gene *ABCC3*; Pattern 3, represented by gene *A2M*; Pattern 1, represented by gene *ACTB*.

**
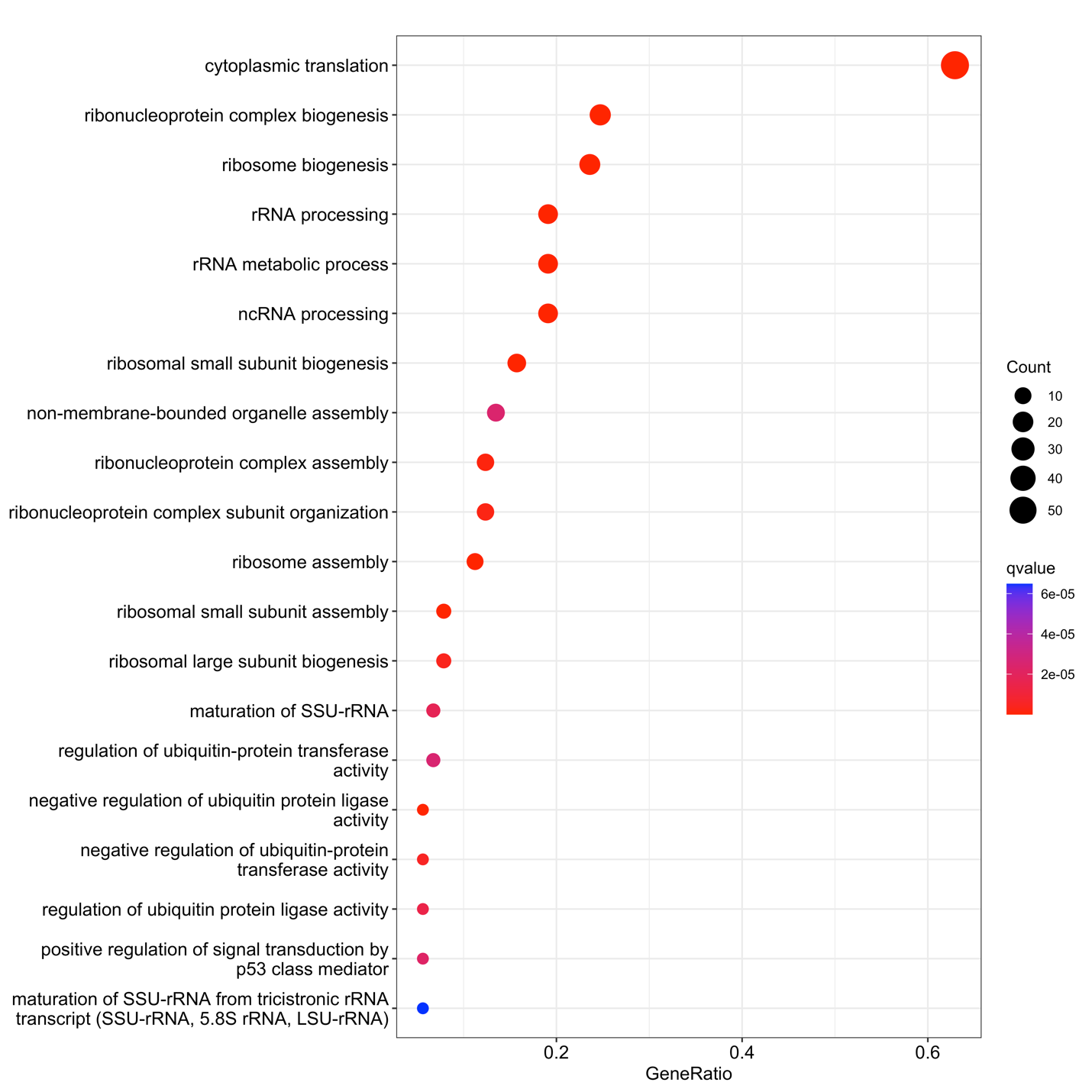
**

**Supplementary Figure 26: Gene Ontology enrichment analysis on SVGs of Pattern 1 in Rheumatoid Arthritis (RA) study using 3D SRT.** P-values are one-sided Fisher's exact test adjusted by FDR.

**
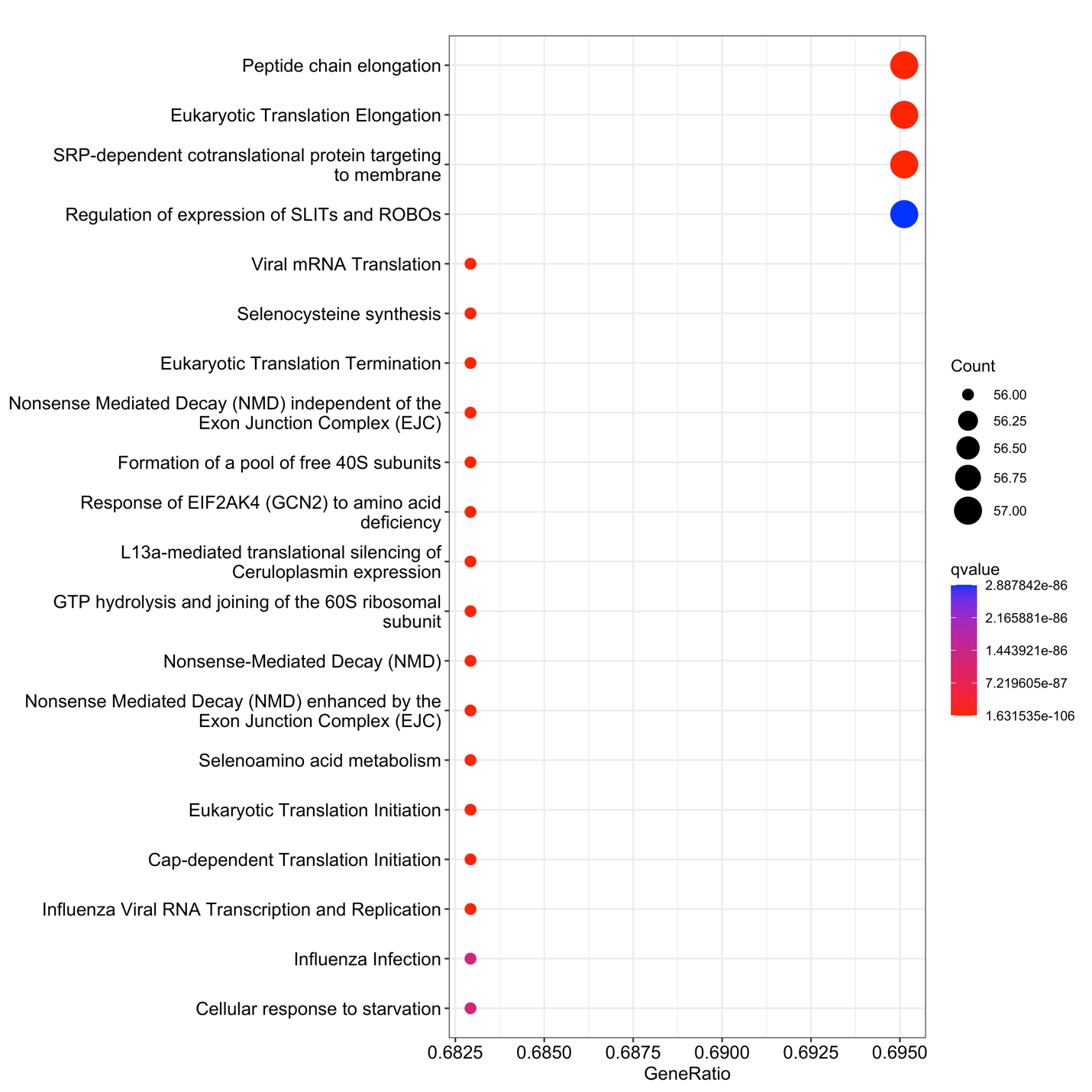
**

**Supplementary Figure 27: Pathway enrichment analysis on SVGs of Pattern 1 in Rheumatoid Arthritis (RA) study using 3D SRT.** P-values are one-sided Fisher's exact test adjusted by FDR.

**
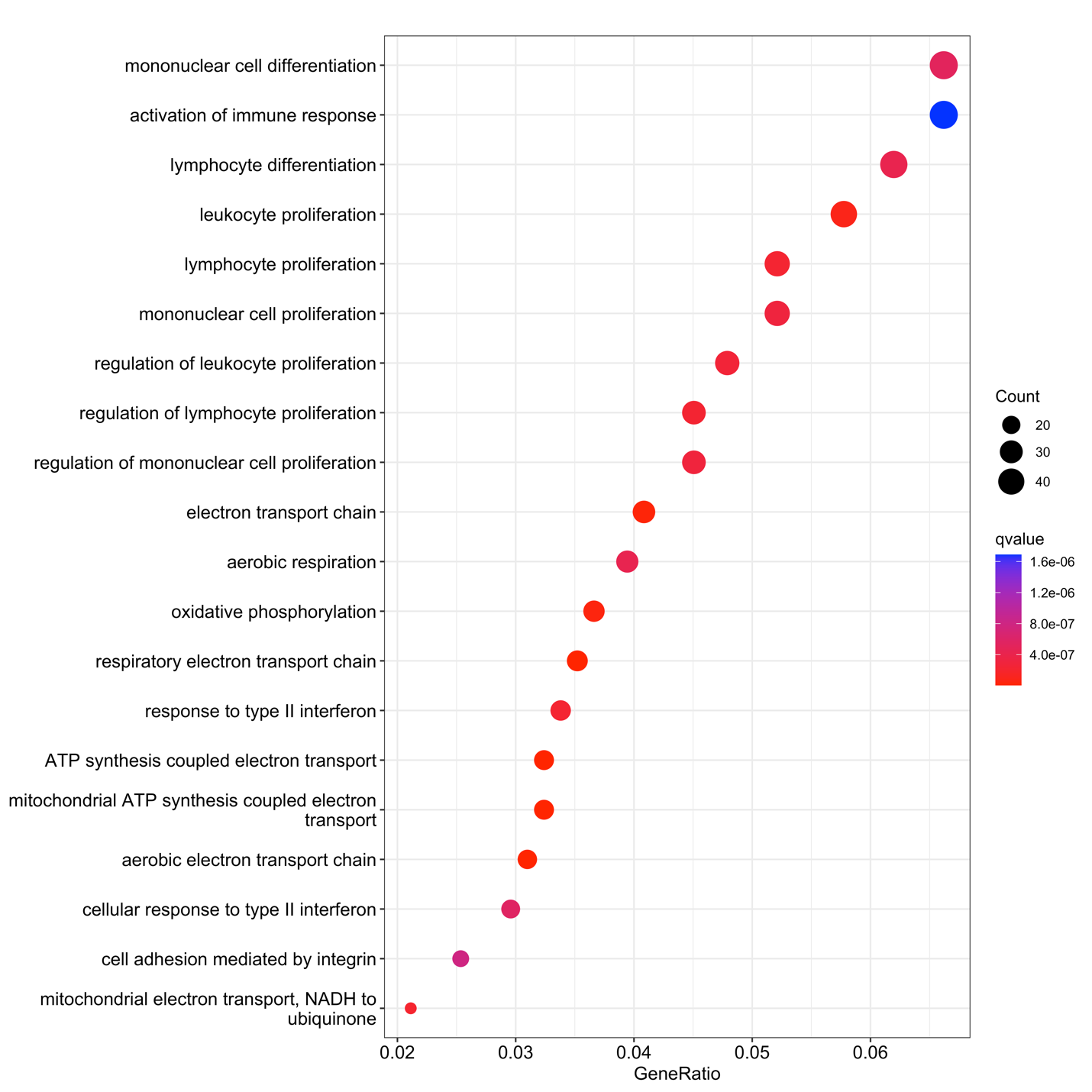
**

**Supplementary Figure 28: Gene Ontology enrichment analysis on SVGs of Pattern 2 in Rheumatoid Arthritis (RA) study using 3D SRT.** P-values are one-sided Fisher's exact test adjusted by FDR.

**
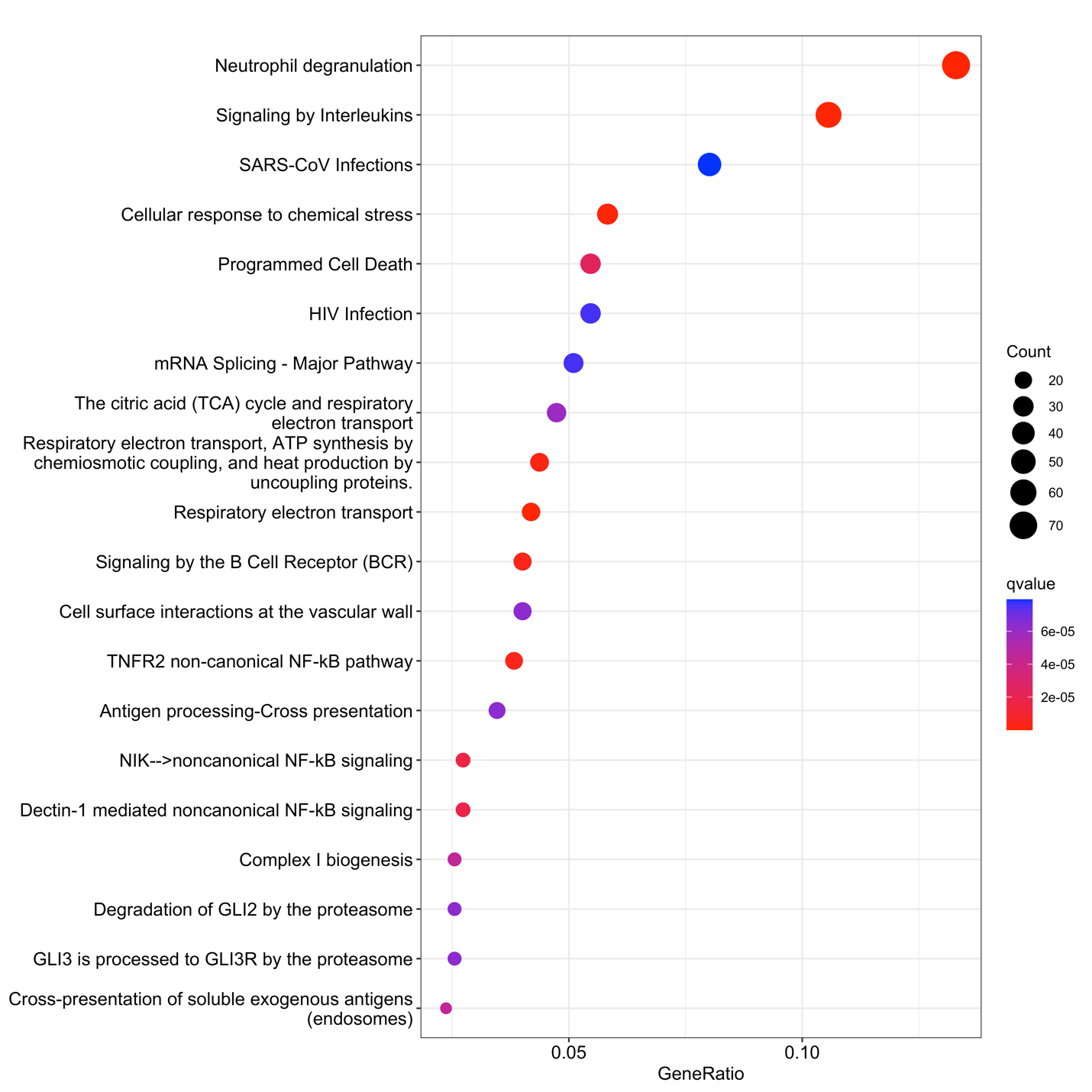
**

**Supplementary Figure 29: Pathway analysis on SVGs of Pattern 2 in Rheumatoid Arthritis (RA) study using 3D SRT.** P-values are one-sided Fisher's exact test adjusted by FDR.

**
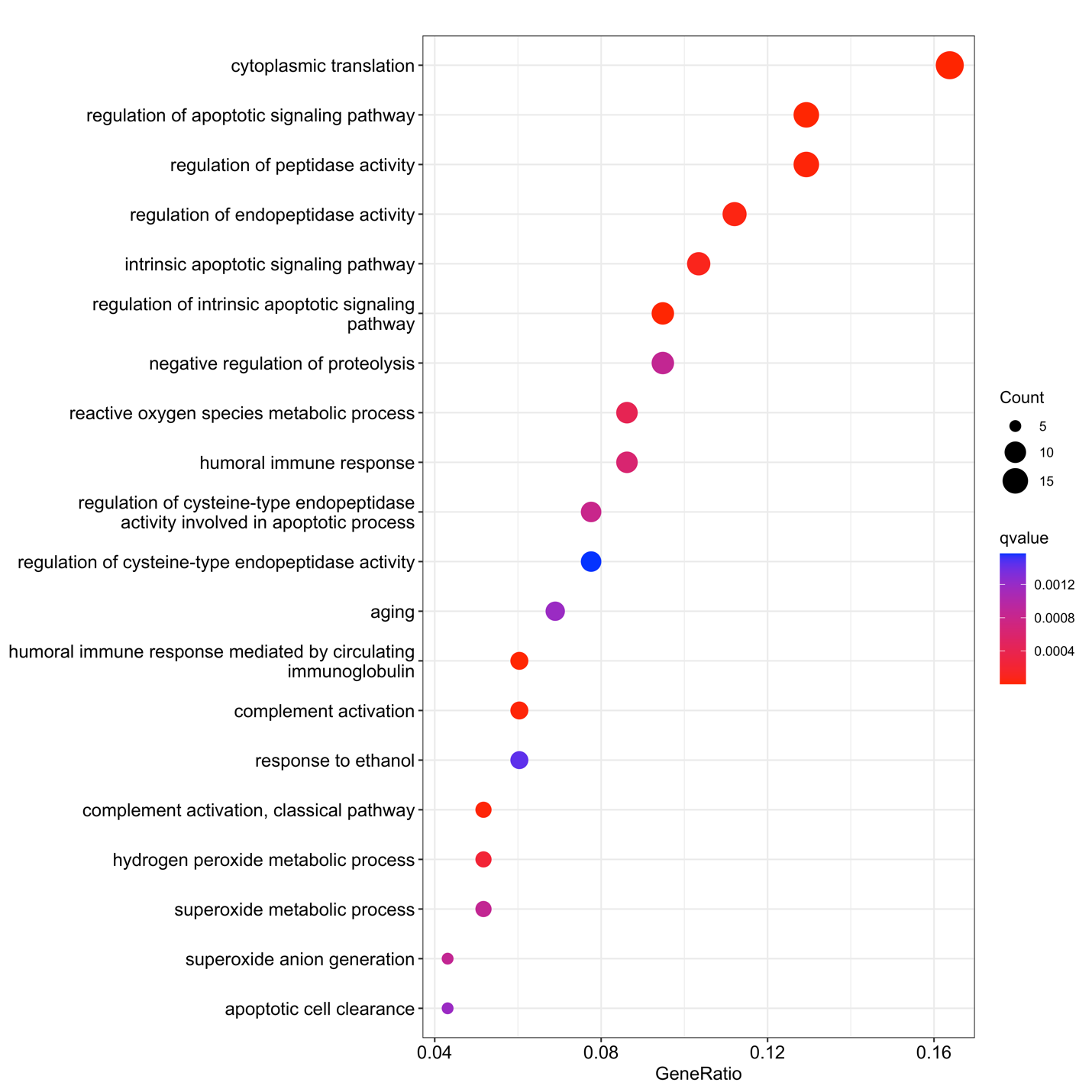
**

**Supplementary Figure 30: Gene Ontology enrichment analysis on SVGs of Pattern 3 in Rheumatoid Arthritis (RA) study using 3D SRT.** P-values are one-sided Fisher's exact test adjusted by FDR.

**
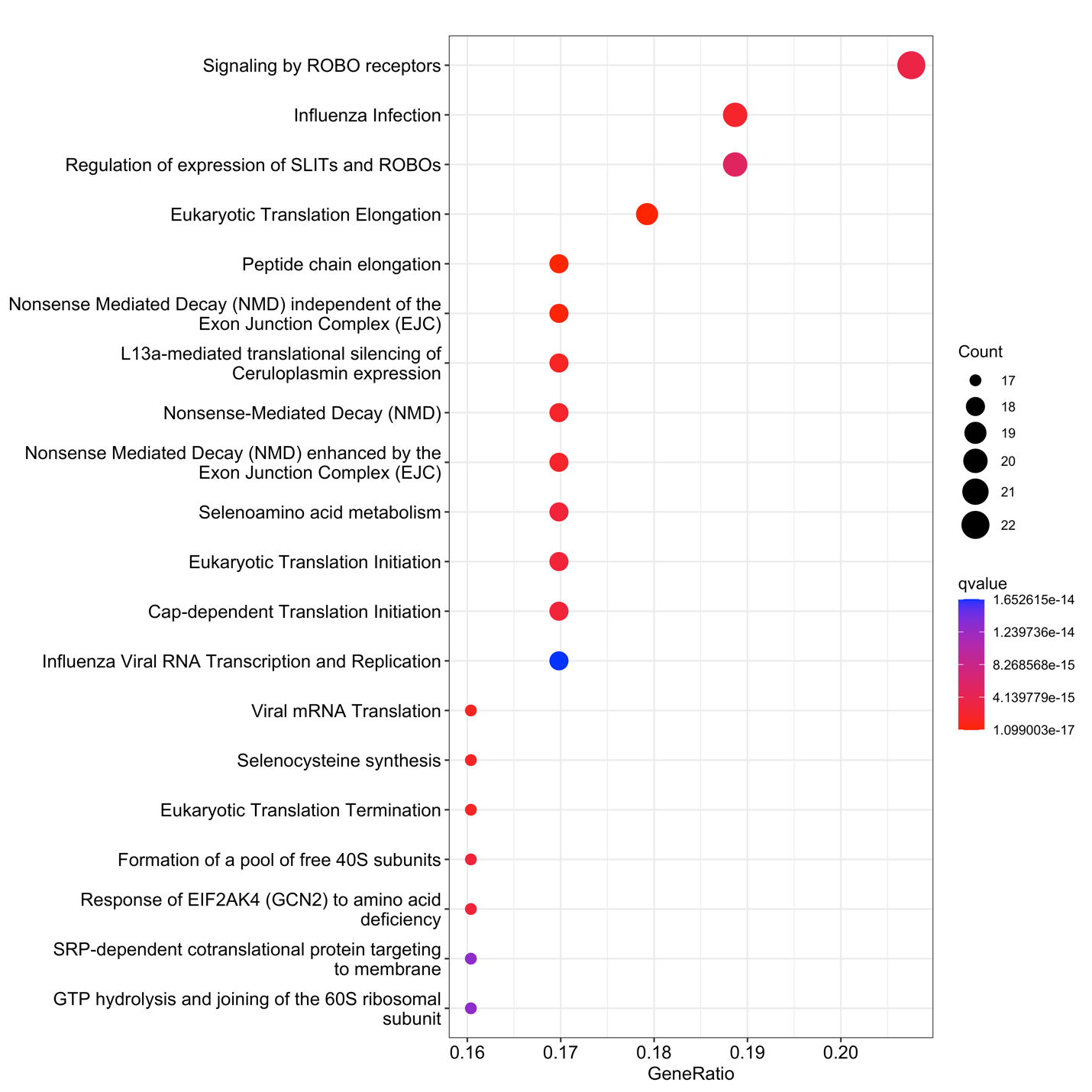
**

**Supplementary Figure 31: Pathway enrichment analysis on SVGs of Pattern 3 in Rheumatoid Arthritis (RA) study using 3D SRT.** P-values are one-sided Fisher's exact test adjusted by FDR.

**
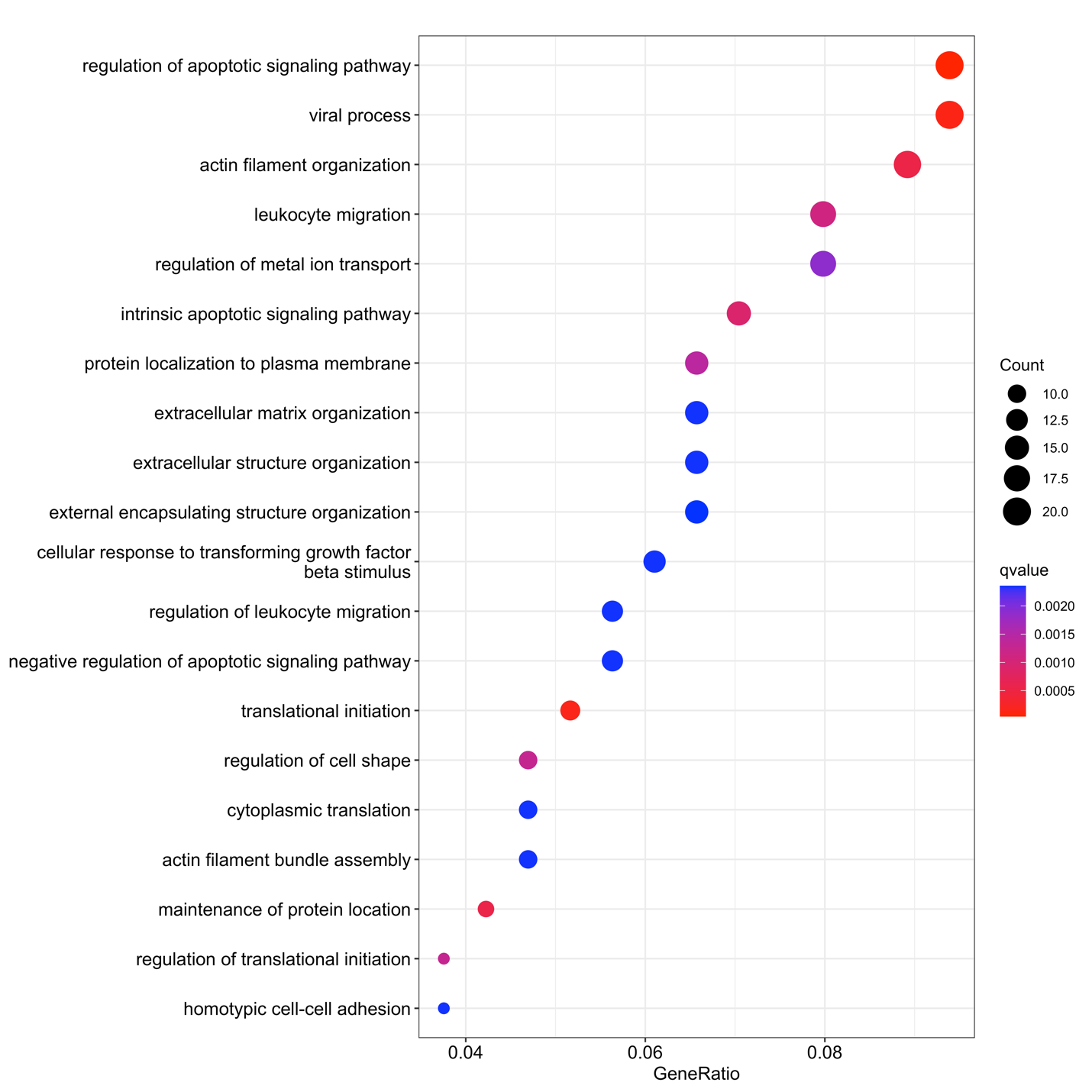
**

**Supplementary Figure 32: Gene Ontology enrichment analysis on SVGs of Pattern 4 in Rheumatoid Arthritis (RA) study using 3D SRT.** P-values are one-sided Fisher's exact test adjusted by FDR.

**
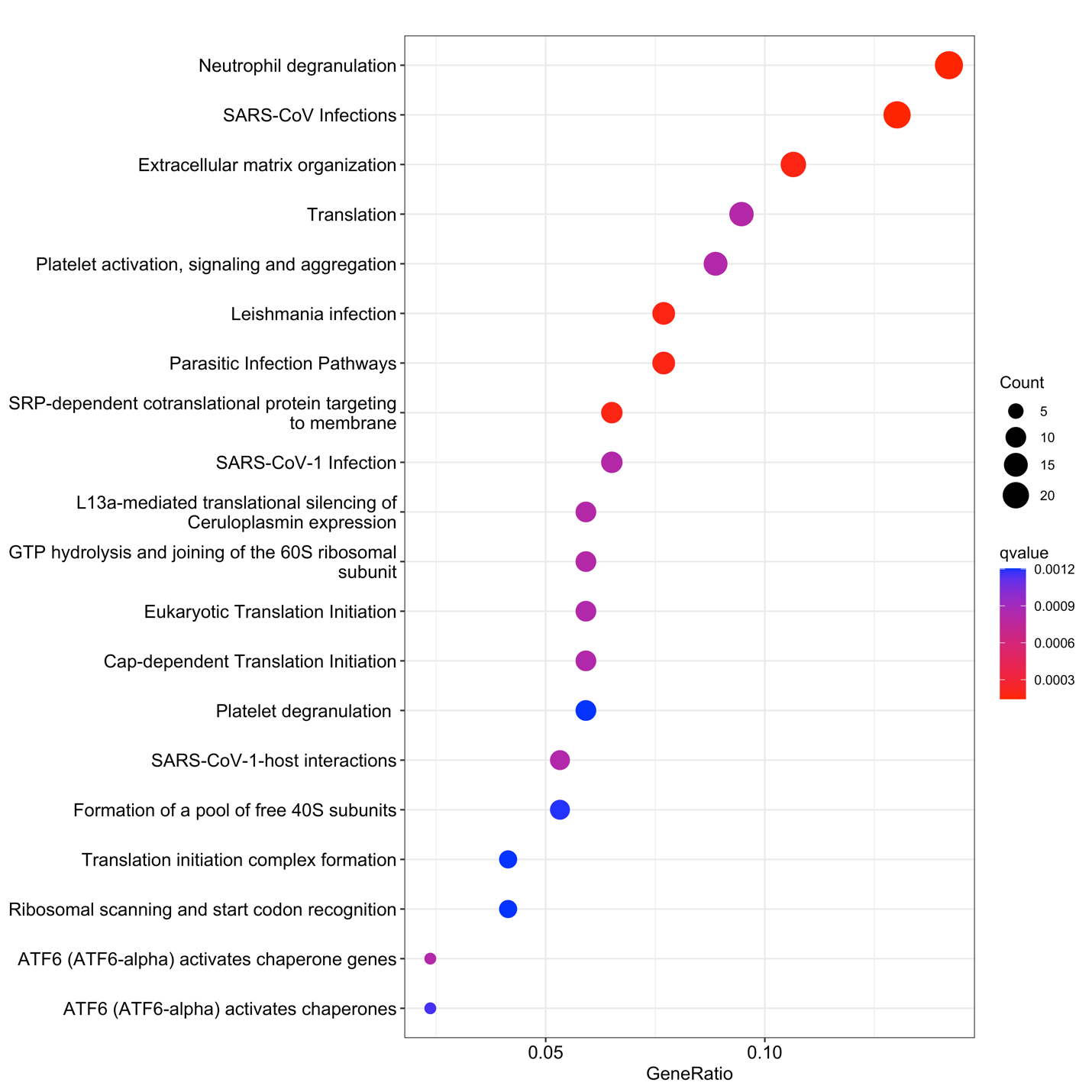
**

**Supplementary Figure 33: Pathway enrichment analysis on SVGs of Pattern 4 in Rheumatoid Arthritis (RA) study using 3D SRT.** P-values are one-sided Fisher's exact test adjusted by FDR.


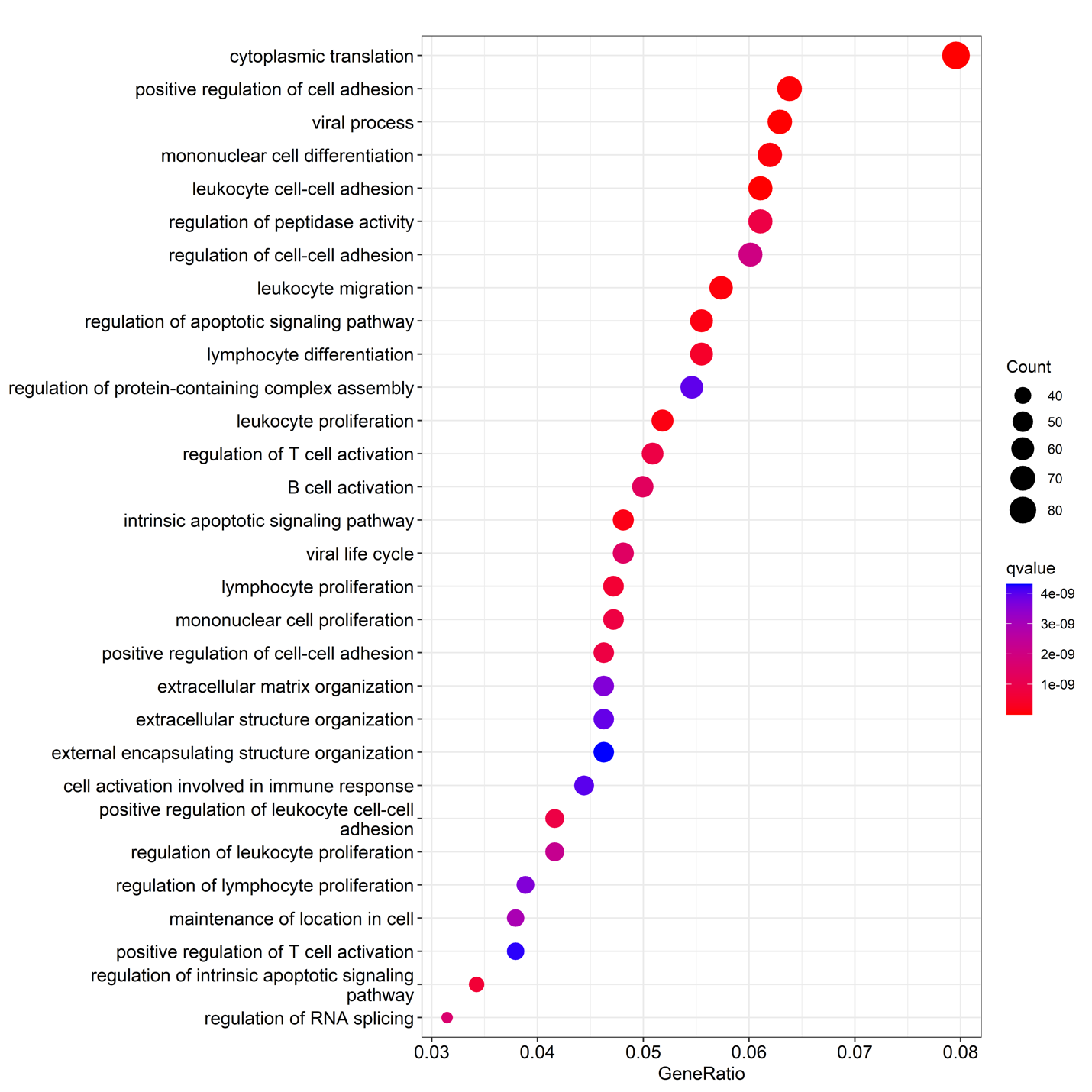


**Supplementary Figure 34: Gene Ontology enrichment analysis on patient RA2.** P-values are one-sided Fisher's exact test adjusted by FDR.


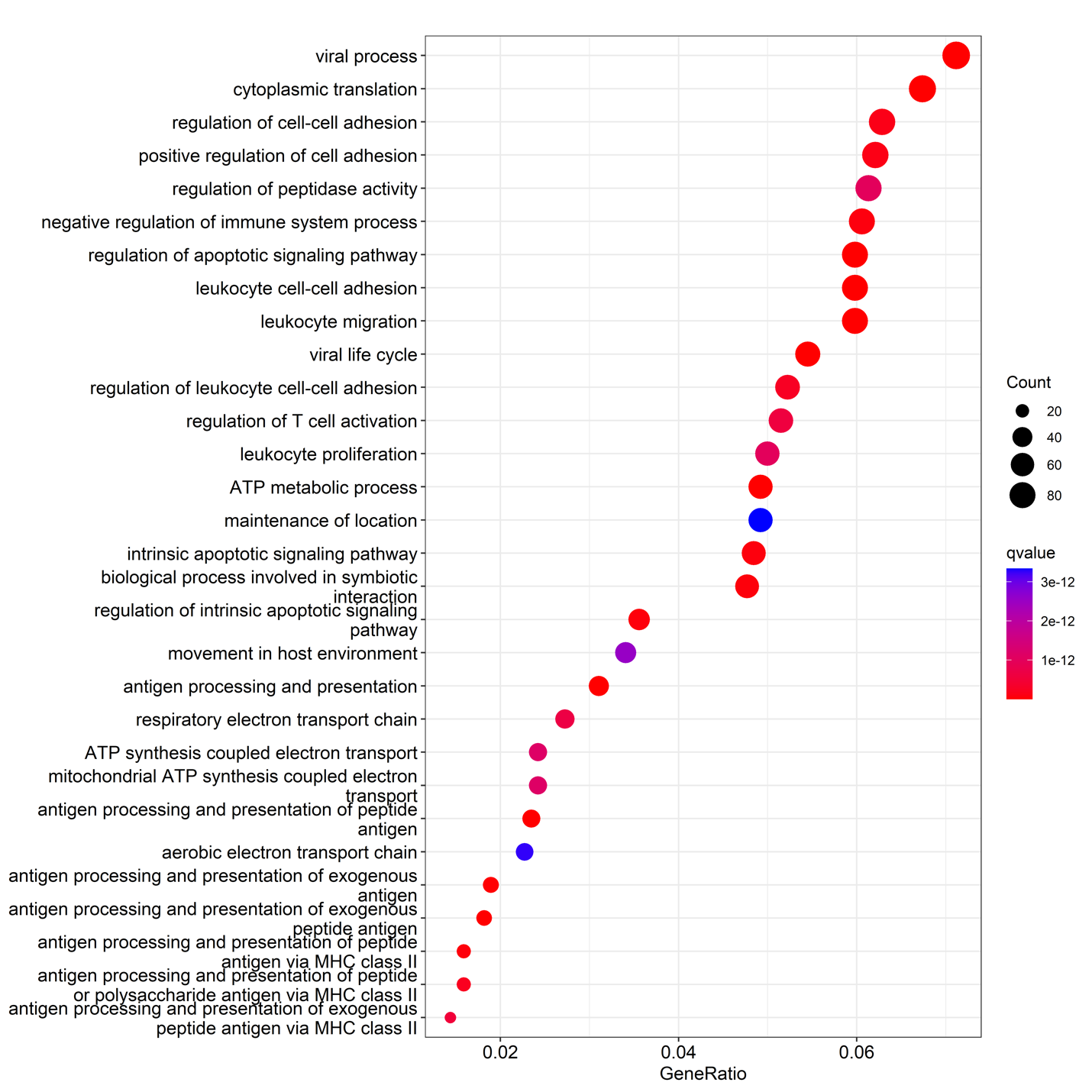


**Supplementary Figure 35: Gene Ontology enrichment analysis on patient RA3.** P-values are one-sided Fisher's exact test adjusted by FDR.

**
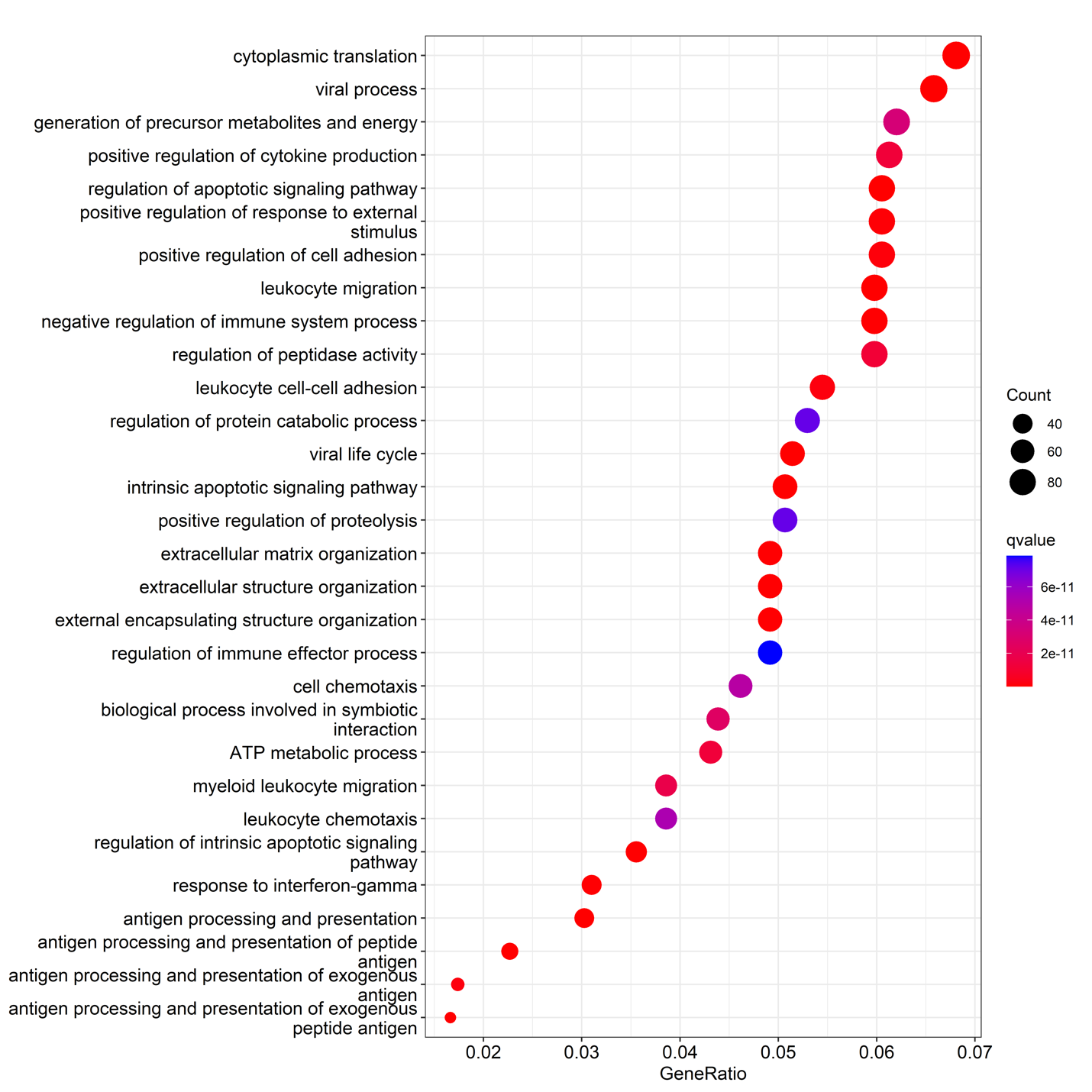
**

**Supplementary Figure 36: Gene Ontology enrichment analysis on patient RA4.** P-values are one-sided Fisher's exact test adjusted by FDR.

**
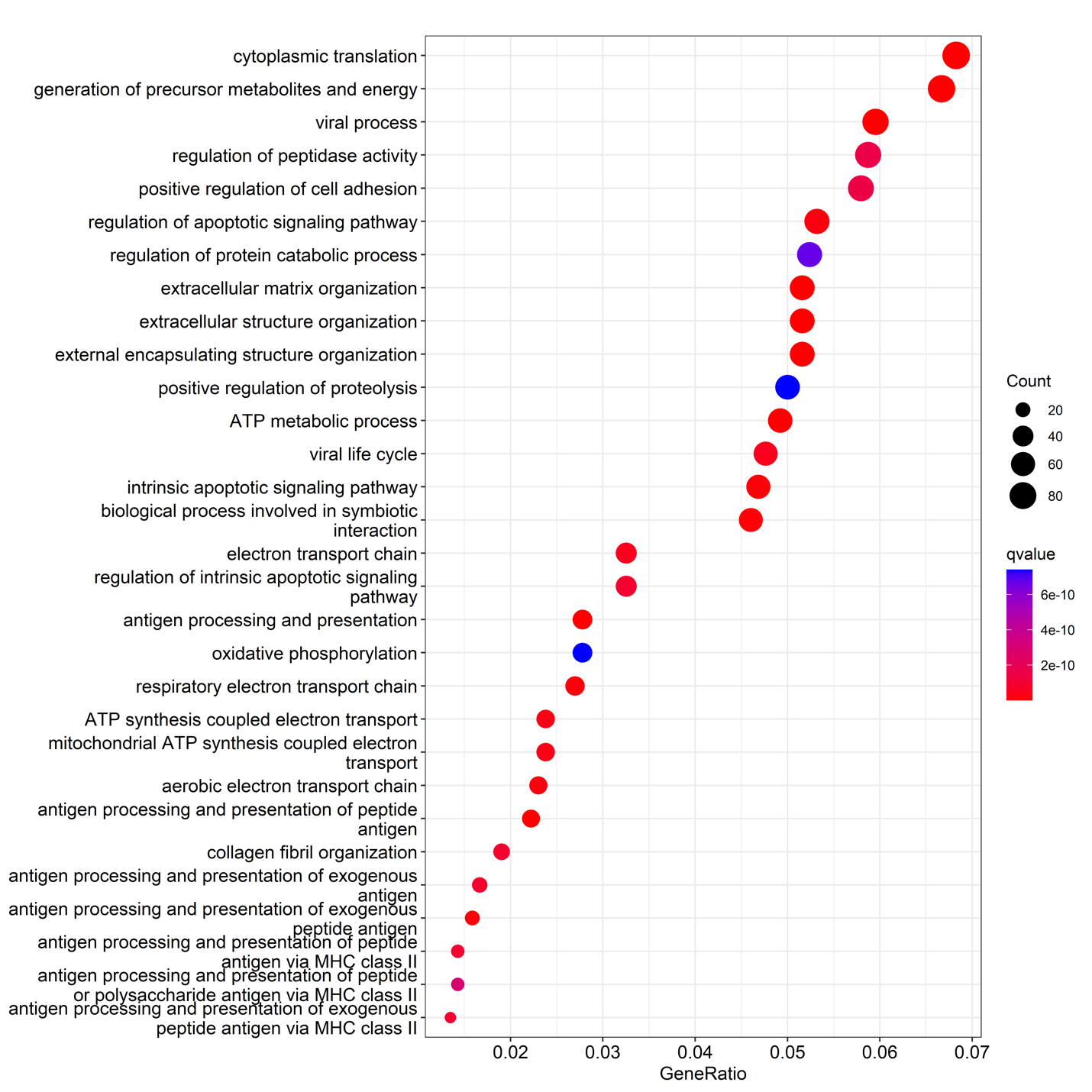
**

**Supplementary Figure 37: Gene Ontology enrichment analysis on patient RA5.** P-values are one-sided Fisher's exact test adjusted by FDR.

**
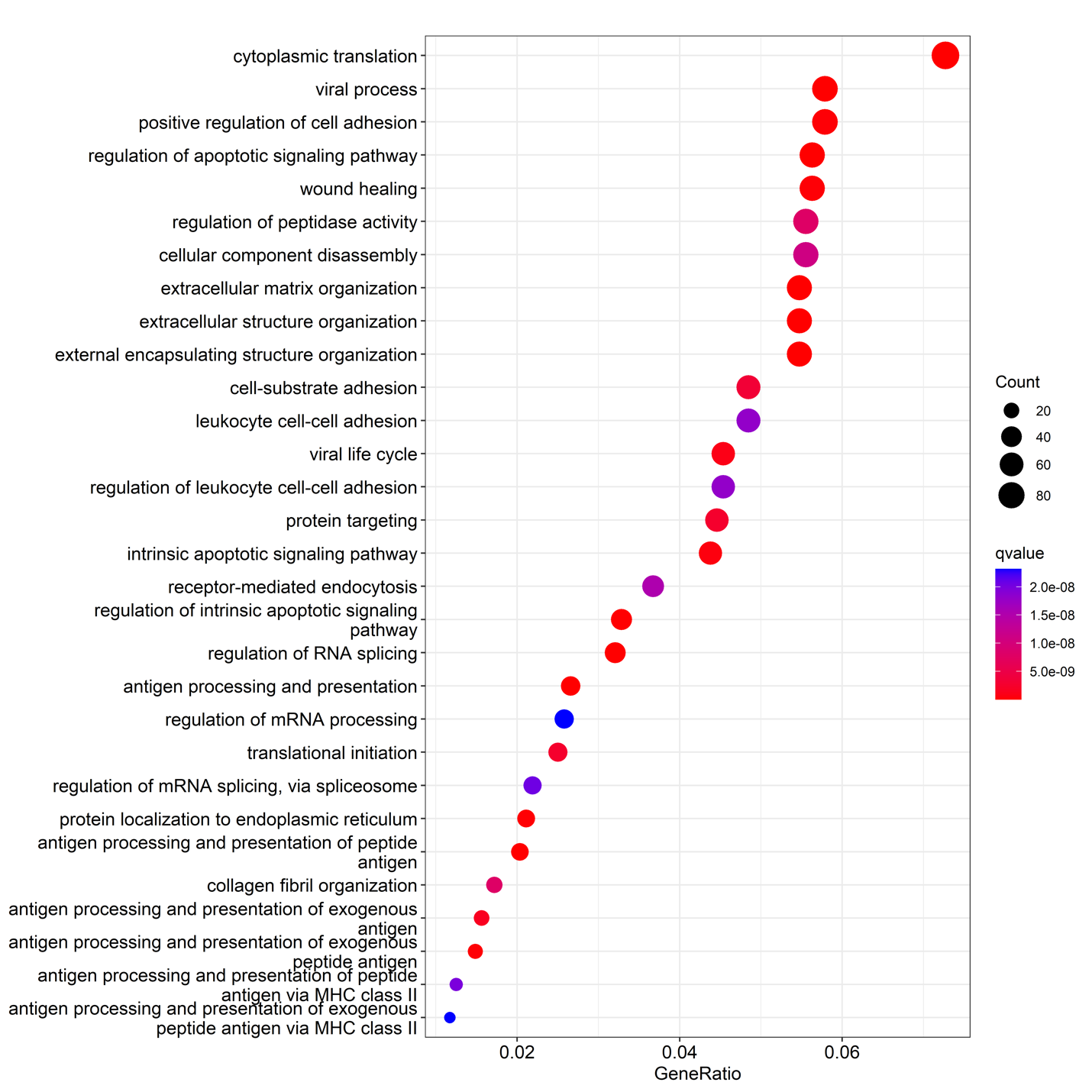
**

**Supplementary Figure 38: Gene Ontology enrichment analysis on patient RA6.** P-values are one-sided Fisher's exact test adjusted by FDR.

**
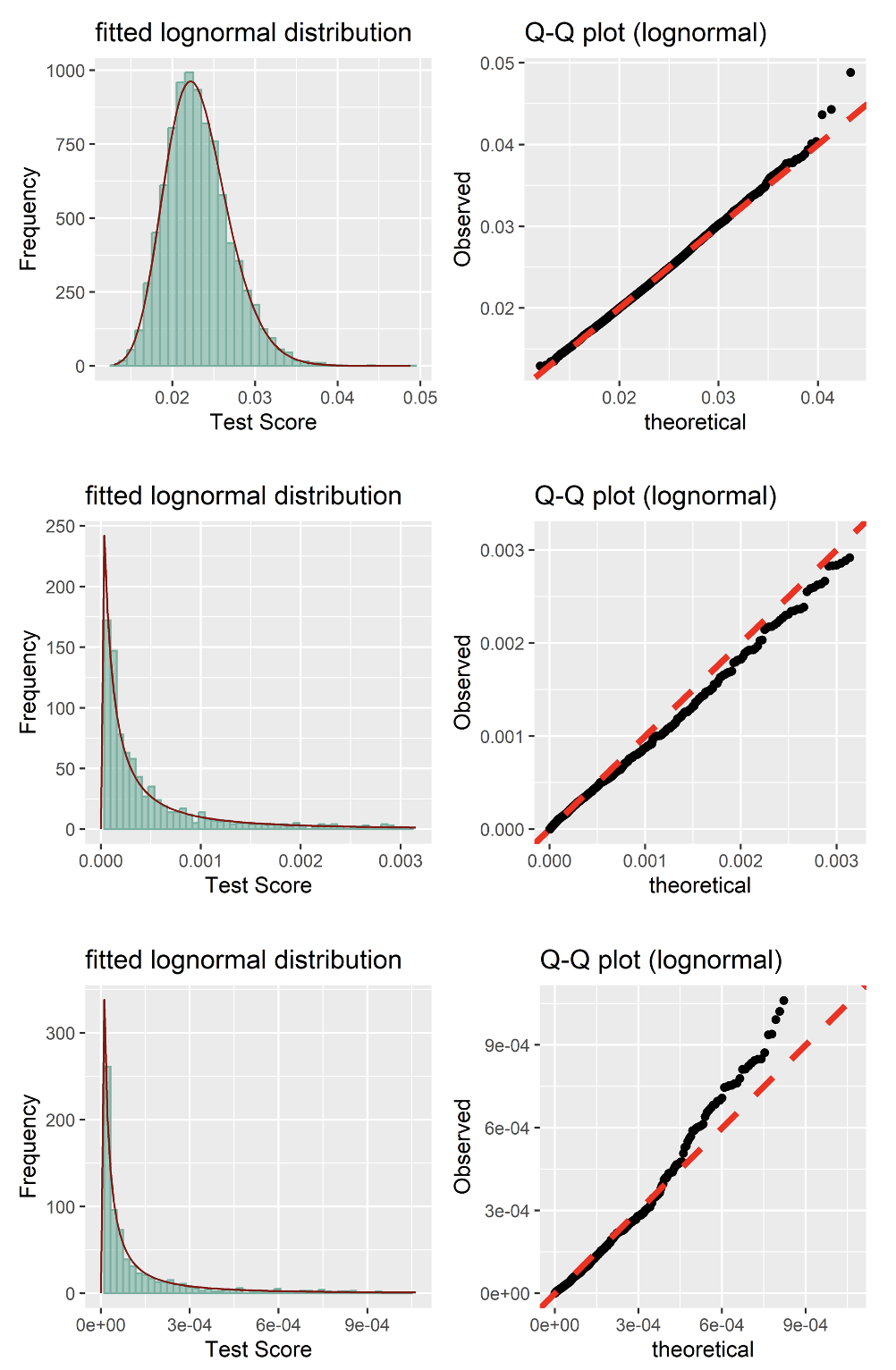
**

**Supplementary Figure 39: Distribution of test scores and fitted lognormal distribution.** Histograms of the test scores on permuted data vs. density of fitted lognormal distribution (left) and corresponding Q-Q plots (right). The permuted data from simulations, mouse olfactory bulb data, and human breast cancer data are shown in the top, middle, and bottom rows, respectively. The simulation (top) is from continuous 3D Pattern I. All three continuous 3D patterns have similar results.


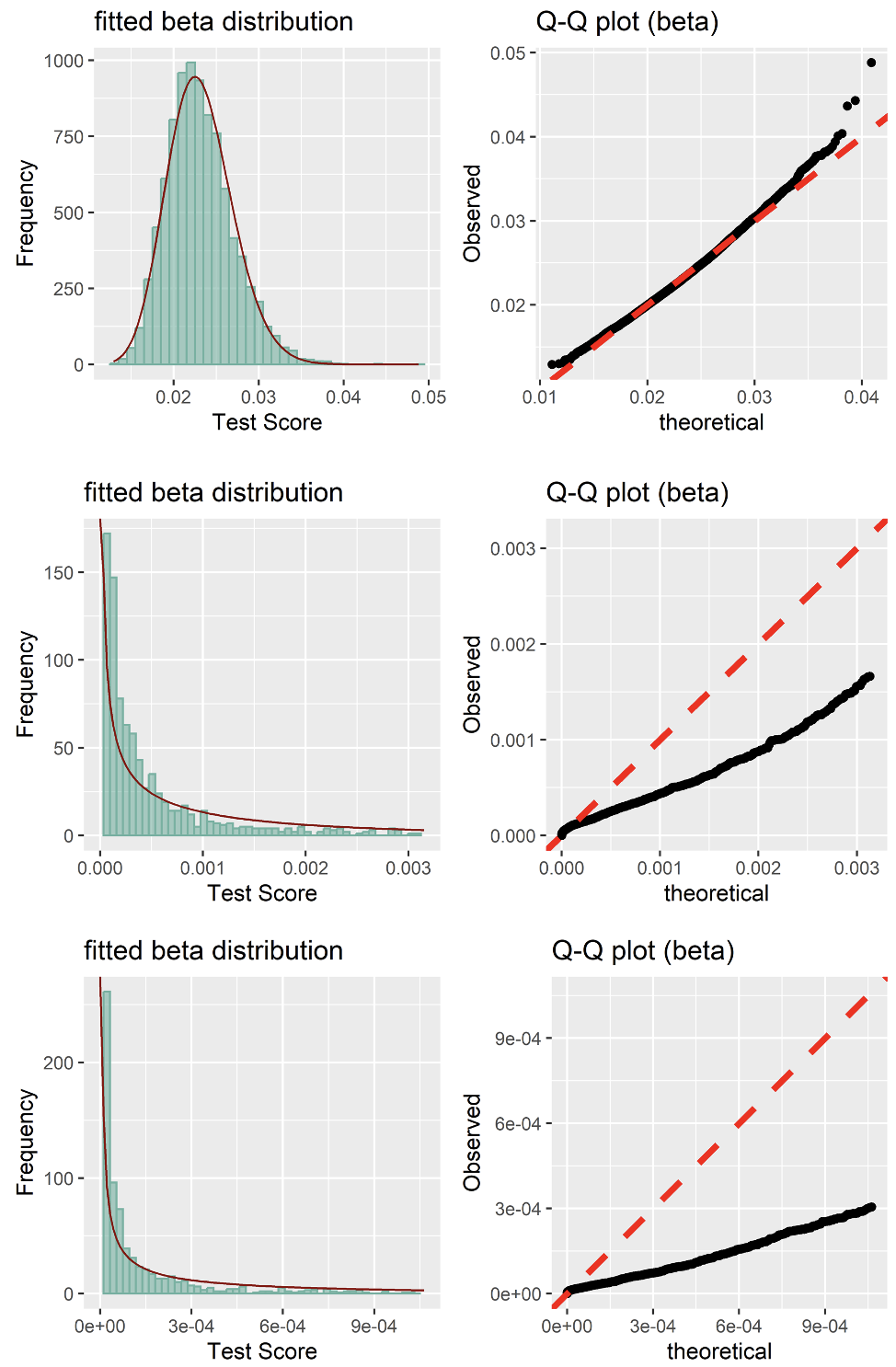


**Supplementary Figure 40: Distribution of test scores and fitted beta distribution.** Histograms of the test scores on permuted data vs. density of fitted beta distribution (left) and corresponding Q-Q plots (right). The permuted data from simulations, mouse olfactory bulb data, and human breast cancer data are shown in the top, middle, and bottom rows, respectively. The simulation (top) is from continuous 3D Pattern I. All three continuous 3D patterns have similar results.


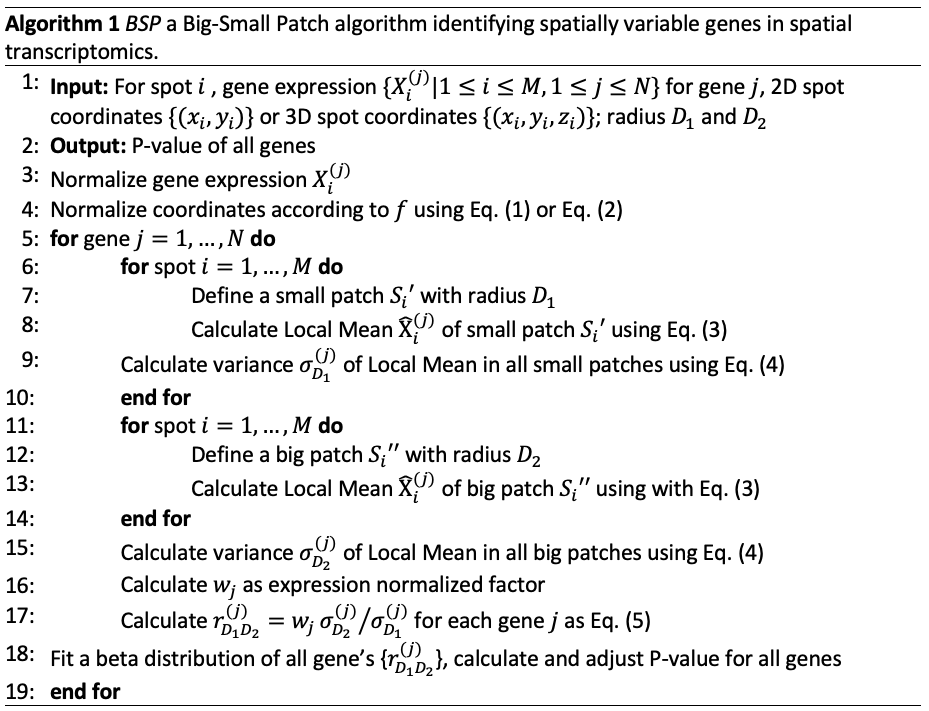


**Supplementary Figure 41: Pseudocode of BSP algorithm.**

**
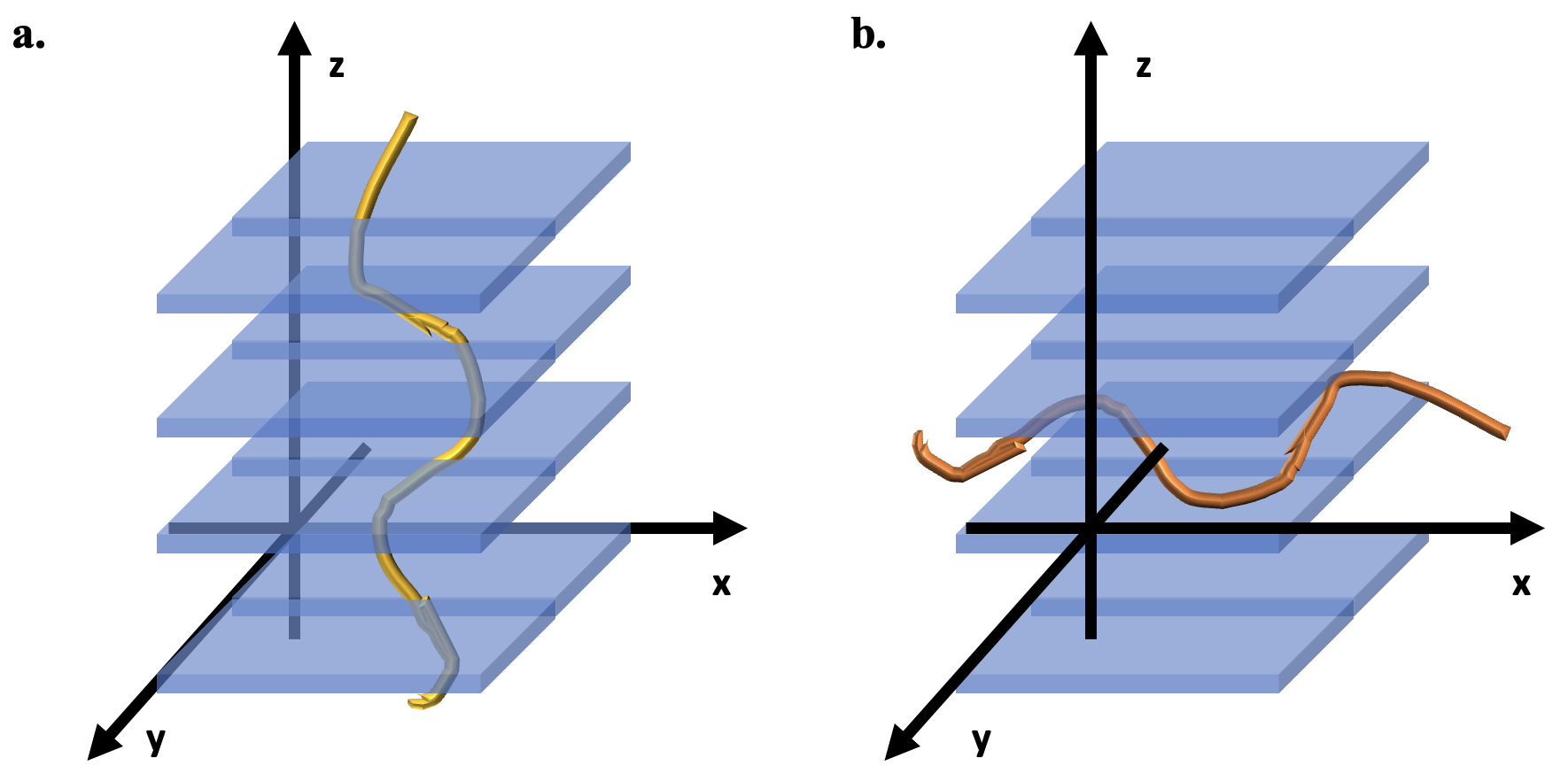
**

**Supplementary Figure 42: 3D simulation strategies. a) Simulation adopted**: Fixed the direction of the random walks in the z-axis to ensure that the sliced planes can capture the simulated spatial pattern. **b) Simulation avoided**: The direction of the random walks may be horizontal with the z-axis, so the simulated 3D spatial pattern cannot be captured by the sliced planes.
