# Supplementary material for: Dimension-agnostic and granularity-based spatially variable gene identification using BSP
Source: Nat Commun. 2023 Nov 14;14:7367. doi: 10.1038/s41467-023-43256-5 (PMC10645821; doi:10.1038/s41467-023-43256-5)
Supplement: Supplementary file 3 — Description of Additional Supplementary Files [file 41467_2023_43256_MOESM3_ESM.pdf]

Description of Additional Supplementary Files:

Supplementary Data 1: Comparison of the computational time and memory on an Ubuntu 16.04.4 LTS workstation with Intel(R) Xeon(R) W-2125 CPU @ 4.00GHz and 32 GB memory

Supplementary Data 2: BSP results on ST Mouse Olfactory Bulb study

Supplementary Data 3: BSP results on ST human breast cancer study

Supplementary Data 4: BSP results on hippocampus study using SeqFISH. The p-values are from one-sided tests without multi-test corrections.

Supplementary Data 5: BSP results on hypothalamus preoptic regions study using MERFISH. The p-values are from one-sided tests without multi-test corrections.

Supplementary Data 6: BSP results on Acute Kidney Injury (AKI) study using 10X Visium. The p-values are from one-sided tests without multi-test corrections.

Supplementary Data 7: GO enrichment analysis on Acute Kidney Injury (AKI) study using 10X Visium. P-values are one-sided Fisher's exact test adjusted by FDR.

Supplementary Data 8: Reactome analysis on Acute Kidney Injury (AKI) study using 10X Visium. P-values are one-sided Fisher's exact test adjusted by FDR.

Supplementary Data 9: Disease Ontology analysis on Acute Kidney Injury (AKI) study using 10X visium. P-values are one-sided Fisher's exact test adjusted by FDR.

Supplementary Data 10: GO enrichment analysis on SVGs of Pattern 1 in Acute Kidney Injury (AKI) study using 10X Visium. P-values are one-sided Fisher's exact test adjusted by FDR.

Supplementary Data 11: KEGG pathway analysis on SVGs of Pattern 1 in Acute Kidney Injury (AKI) study using 10X Visium. P-values are one-sided Fisher's exact test adjusted by FDR.

Supplementary Data 12: GO enrichment analysis on SVGs of Pattern 2 in Acute Kidney Injury (AKI) study using 10X Visium. P-values are one-sided Fisher's exact test adjusted by FDR.

Supplementary Data 13: KEGG pathway analysis on SVGs of Pattern 2 in Acute Kidney Injury (AKI) study using 10X Visium. P-values are one-sided Fisher's exact test adjusted by FDR.

Supplementary Data 14: BSP computational resource analysis

Supplementary Data 15: PanglaoDB results on Slide-seq V1 mouse cerebellum study. P-values are one-sided Fisher's exact test without multi-test corrections.

Supplementary Data 16: PanglaoDB results on Slide-seq V2 mouse cerebellum study. P-values are one-sided Fisher's exact test without multi-test corrections.

Supplementary Data 17: PanglaoDB results on HDST olfactory bulb study. P-values are one-sided Fisher's exact test without multi-test corrections.

Supplementary Data 18: Methods comparision on Slide-seq V2 mouse cerebellum study. The p-values were from one-sided tests without multi-test corrections.

Supplementary Data 19: GO enrichment analysis on identified SVGs in Slide-seq V2 mouse cerebellum study. P-values are one-sided Fisher's exact test adjusted by FDR.

Supplementary Data 20: 724 genes detected both by 2D and 3D data using BSP on patient RA1. The p-values are from one-sided tests without multi-test corrections.

Supplementary Data 21: 532 genes detected only by 3D but failed by 2D meta-analysis using BSP on patient RA1. The p-values are from one-sided tests without multi-test corrections.

Supplementary Data 22: 80 genes detected only by 2D meta-analysis but failed by 3D using BSP on patient RA1. The p-values are from one-sided tests without multi-test corrections.

Supplementary Data 23: Gene ontology enrichment analysis on 1,257 genes identified by 3D settings in patient RA1. P-values are one-sided Fisher's exact test adjusted by FDR.

Supplementary Data 24: Gene ontology enrichment analysis on 724 genes identified by both 2D meta-analysis and 3D settings in patient RA1. P-values are one-sided Fisher's exact test adjusted by FDR.

Supplementary Data 25: Gene ontology enrichment analysis on 532 genes identified only by 3D settings in patient RA1. P-values are one-sided Fisher's exact test adjusted by FDR.

Supplementary Data 26: Gene Ontology enrichment analysis on SVGs of Pattern 1 in Rheumatoid Arthritis (RA) study using 3D SRT. P-values are one-sided Fisher's exact test adjusted by FDR.

Supplementary Data 27: Pathway enrichment analysis on SVGs of Pattern 1 in Rheumatoid Arthritis (RA) study using 3D SRT. P-values are one-sided Fisher's exact test adjusted by FDR.

Supplementary Data 28: Gene Ontology enrichment analysis on SVGs of Pattern 2 in Rheumatoid Arthritis (RA) study using 3D SRT. P-values are one-sided Fisher's exact test adjusted by FDR.

Supplementary Data 29: Pathway enrichment analysis on SVGs of Pattern 2 in Rheumatoid Arthritis (RA) study using 3D SRT. P-values are one-sided Fisher's exact test adjusted by FDR.

Supplementary Data 30: Gene Ontology enrichment analysis on SVGs of Pattern 3 in Rheumatoid Arthritis (RA) study using 3D SRT. P-values are one-sided Fisher's exact test adjusted by FDR.

Supplementary Data 31: Pathway enrichment analysis on SVGs of Pattern 3 in Rheumatoid Arthritis (RA) study using 3D SRT. P-values are one-sided Fisher's exact test adjusted by FDR.

Supplementary Data 32: Gene Ontology enrichment analysis on SVGs of Pattern 4 in Rheumatoid Arthritis (RA) study using 3D SRT. P-values are one-sided Fisher's exact test adjusted by FDR.

Supplementary Data 33: Pathway enrichment analysis on SVGs of Pattern 4 in Rheumatoid Arthritis (RA) study using 3D SRT. P-values are one-sided Fisher's exact test adjusted by FDR.

Supplementary Data 34: Goodness-of-fit test with Cramer-von Mises criterion on lognormal distribution. The p-values are from one-sided tests without multi-test corrections.

Supplementary Data 35: Goodness-of-fit test with Cramer-von Mises criterion on beta distribution. The p-values are from one-sided tests without multi-test corrections.

Supplementary Data 36: Selection D2 on identifying SVGs on real data of mouse olfactory bulb and human breast cancer
