# Supplementary material for: Dimension-agnostic and granularity-based spatially variable gene identification using BSP
Source: Nat Commun. 2023 Nov 14;14:7367. doi: 10.1038/s41467-023-43256-5 (PMC10645821; doi:10.1038/s41467-023-43256-5)
Supplement: Supplementary file 5 — Reporting Summary [file 41467_2023_43256_MOESM5_ESM.pdf]

nature portfolio

Corresponding author(s): Juexin Wang, Qin Ma, and Dong Xu

Last updated by author(s): Oct 19, 2023

# Reporting Summary

Nature Portfolio wishes to improve the reproducibility of the work that we publish. This form provides structure for consistency and transparency in reporting. For further information on Nature Portfolio policies, see our Editorial Policies and the Editorial Policy Checklist.

## Statistics

For all statistical analyses, confirm that the following items are present in the figure legend, table legend, main text, or Methods section.

| n/a | Confirmed | |
|---|---|---|
| ☐ | ☒ | The exact sample size (*n*) for each experimental group/condition, given as a discrete number and unit of measurement |
| ☐ | ☒ | A statement on whether measurements were taken from distinct samples or whether the same sample was measured repeatedly |
| ☐ | ☒ | The statistical test(s) used AND whether they are one- or two-sided<br>*Only common tests should be described solely by name; describe more complex techniques in the Methods section.* |
| ☐ | ☒ | A description of all covariates tested |
| ☐ | ☒ | A description of any assumptions or corrections, such as tests of normality and adjustment for multiple comparisons |
| ☐ | ☒ | A full description of the statistical parameters including central tendency (e.g. means) or other basic estimates (e.g. regression coefficient) AND variation (e.g. standard deviation) or associated estimates of uncertainty (e.g. confidence intervals) |
| ☐ | ☒ | For null hypothesis testing, the test statistic (e.g. *F*, *t*, *r*) with confidence intervals, effect sizes, degrees of freedom and *P* value noted<br>*Give P values as exact values whenever suitable.* |
| ☒ | ☐ | For Bayesian analysis, information on the choice of priors and Markov chain Monte Carlo settings |
| ☒ | ☐ | For hierarchical and complex designs, identification of the appropriate level for tests and full reporting of outcomes |
| ☒ | ☐ | Estimates of effect sizes (e.g. Cohen's *d*, Pearson's *r*), indicating how they were calculated |

*Our web collection on statistics for biologists contains articles on many of the points above.*

## Software and code

Policy information about availability of computer code

| | |
|---|---|
| Data collection | No software was used |
| Data analysis | BSP is open source and publicly available at https://github.com/juexinwang/bsp. Other tools and packages used in this paper include: Python 3.9.12, scikit-learn version 1.1.2, numpy version 1.23.3, pandas version 1.5.0, topGO version 3.16, ReactomePA version 3.16, DOSE version 3.16, rPanglaoDB version 0.2.1 |

For manuscripts utilizing custom algorithms or software that are central to the research but not yet described in published literature, software must be made available to editors and reviewers. We strongly encourage code deposition in a community repository (e.g. GitHub). See the Nature Portfolio guidelines for submitting code & software for further information.

## Data

Policy information about availability of data

All manuscripts must include a data availability statement. This statement should provide the following information, where applicable:

- Accession codes, unique identifiers, or web links for publicly available datasets
- A description of any restrictions on data availability
- For clinical datasets or third party data, please ensure that the statement adheres to our policy

The mouse olfactory bulb and human breast cancer data are available at http://www.spatialtranscriptomicsresearch.org, the MERFISH data can be downloaded from (https://datadryad.org/stash/dataset/doi:10.5061/dryad.8t8s248), and the SeqFISH data is available at https://www.cell.com/cms/10.1016/j.neuron.2016.10.001/attachment/759be4dc-04a6-4a58-b6f6-9b52be2802db/mmc6.xlsx. Slide-seq data, Slide-seqV2 data, HDST data, and human rheumatoid

arthritis synovium data are available at Broad Institute's single-cell repository (https://singlecell.broadinstitute.org/single_cell/) with ID SCP354, SCP948, SCP420, and SCP1414. The STARmap data set is available at https://github.com/drieslab/spatial-datasets/tree/master/data/2018_starmap_3D_cortex. The kidney spatial transcriptomics data can be downloaded from the Kidney Tissue Atlas (https://atlas.kpmp.org/) with ID 32-10074. The generated simulation data has been deposited in Figshare database at (https://doi.org/10.6084/m9.figshare.24187923). Source data are provided with this paper.

## Research involving human participants, their data, or biological material

Policy information about studies with human participants or human data. See also policy information about sex, gender (identity/presentation), and sexual orientation and race, ethnicity and racism.

| | |
|---|---|
| Reporting on sex and gender | Not applicable. |
| Reporting on race, ethnicity, or other socially relevant groupings | Not applicable. |
| Population characteristics | Not applicable. |
| Recruitment | Not applicable. |
| Ethics oversight | Not applicable. |

Note that full information on the approval of the study protocol must also be provided in the manuscript.

# Field-specific reporting

Please select the one below that is the best fit for your research. If you are not sure, read the appropriate sections before making your selection.

☒ Life sciences ☐ Behavioural & social sciences ☐ Ecological, evolutionary & environmental sciences

For a reference copy of the document with all sections, see nature.com/documents/nr-reporting-summary-flat.pdf

# Life sciences study design

All studies must disclose on these points even when the disclosure is negative.

| | |
|---|---|
| Sample size | No data collection was involved in the present study. All sample size determination information, if available, were described in the original paper for the used data sets. |
| Data exclusions | No data exclusion was involved in the present study. All data exclusion criteria, if applicable, were described in the original paper for the used data set |
| Replication | We did not perform replication. Instead, we cross-validate the findings of the present study by comparing to other published molecular biology results. |
| Randomization | No data collection was involved in the present study. All blinding information, if available, were described in the original paper for the used data sets. |
| Blinding | No data collection was involved in the present study. All blinding information, if available, were described in the original paper for the used data sets. |

# Reporting for specific materials, systems and methods

We require information from authors about some types of materials, experimental systems and methods used in many studies. Here, indicate whether each material, system or method listed is relevant to your study. If you are not sure if a list item applies to your research, read the appropriate section before selecting a response.

## Materials & experimental systems

| n/a | Involved in the study | |
|---|---|---|
| ☒ | ☐ | Antibodies |
| ☒ | ☐ | Eukaryotic cell lines |
| ☒ | ☐ | Palaeontology and archaeology |
| ☒ | ☐ | Animals and other organisms |
| ☒ | ☐ | Clinical data |
| ☒ | ☐ | Dual use research of concern |
| ☒ | ☐ | Plants |

## Methods

| n/a | Involved in the study | |
|---|---|---|
| ☒ | ☐ | ChIP-seq |
| ☒ | ☐ | Flow cytometry |
| ☒ | ☐ | MRI-based neuroimaging |
